# Influence of Marination with Aromatic Herbs and Cold Pressed Oils on Black Angus Beef Meat

**DOI:** 10.3390/foods10092012

**Published:** 2021-08-27

**Authors:** Vasile-Gheorghe Vişan, Maria Simona Chiş, Adriana Păucean, Vlad Mureșan, Andreea Pușcaș, Laura Stan, Dan Cristian Vodnar, Francisc Vasile Dulf, Dorin Țibulcă, Bogdan Alin Vlaic, Iulian Eugen Rusu, Csaba Balasz Kadar, Augustin Vlaic

**Affiliations:** 1 Department of Fundamental Sciences, Faculty of Animal Science and Biotechnologies Cluj-Napoca, University of Agricultural Sciences and Veterinary Medicine of Cluj-Napoca, 3–5 Mănăştur Street, 400372 Cluj-Napoca, Romania; gelu.visan@yahoo.com (V.-G.V.); bogdan.vlaic@usamvcluj.ro (B.A.V.); vlaic.augustin@gmail.com (A.V.); 2 Department of Food Engineering, Faculty of Food Science and Technology, University of Agricultural Sciences and Veterinary Medicine of Cluj-Napoca, 3–5 Mănăştur Street, 400372 Cluj-Napoca, Romania; adriana.paucean@usamvcluj.ro (A.P.); vlad.muresan@usamvcluj.ro (V.M.); andreea.puscas@usamvcluj.ro (A.P.); laurastan@usamvcluj.ro (L.S.); dorin.tibulca@usamvcluj.ro (D.Ț.); iulian.rusu@usamvcluj.ro (I.E.R.); balasz-csaba@usamvcluj.ro (C.B.K.); 3 Institute of Life Sciences, University of Agricultural Sciences and Veterinary Medicine of Cluj-Napoca, 3–5 Mănăștur Street, 400372 Cluj-Napoca, Romania; dan.vodnar@usamvcluj.ro; 4 Department of Environmental and Plant Protection, Faculty of Agriculture, University of Agricultural Sciences and Veterinary Medicine of Cluj-Napoca, 3–5 Mănăştur Street, 400372 Cluj-Napoca, Romania; francisc.dulf@usamvcluj.ro

**Keywords:** marinated beef, GC/MS, HPLC-RID, polyphenols, flavonoids, hedonic test, beef texture

## Abstract

Beef aging is one of the most common methods used for improving its qualities. The main goal of the present study was to analyse the influence of different cold pressed oils and aromatic herbs during marination process on the nutritional, textural, and sensory attributes of the final grilled sirloin samples. In order to fulfil this goal, methods like GC-MS, HPLC/DAD/ESI-MS, HLPC-RID were performed to quantify fatty acids, phenolic acids, and organic acids, respectively. Textural and sensory analysis were performed with CT 3 Texture Analyser and hedonic test. The results showed high improvement of the meat grilled samples regarding the content of phenolic acids, and textural and sensory characteristics. Pearson values indicate strong positive correlations between raw and grilled samples regarding their content in phenolic acids. Hardness, chewiness, gumminess decreased during marination, meanwhile, resilience, and cohesiveness increased. Sensory analysis highlighted that meat samples marinated with olive oil and rosemary for 120 h reached the highest hedonic score among the tested samples.

## 1. Introduction

About 47% of the world’s beef production is produced by European Union, Brazil and the United States. The world food meat consumption in 2030 is estimated to be 45.3 kg per capita and the consumers are demanding increasingly higher quality [1]. It was reported by FAO that world bovine meat output in 2020 will decrease by 1.4% compared with 2019, mainly in Asia, followed by Oceania, South America, Europe and North America. In contrast, despite the impact of the COVID-19 outbreak on the foodservice industry, the bovine meat imports increased in the United States of America, China, and Canada [2]. Beef meat is mostly consumed due to its flavour and the complex composition of macronutrients, the balanced composition of amino acids, vitamins from B group, and high contents of iron, zinc, and phosphorus [3,4]. The USDA Food Composition database indicates the following nutritional values for a raw beef top sirloin [4] meat with acceptable quality: an energy value of 201 (kcal)/100 g and 20.30 g proteins, 12.71 g fat (of which 5.127 g saturated fats and 75 mg cholesterol), 1.05 μg Vitamin B12, and the following amounts of minerals: 52.00 mg Na, 1.48 mg Fe, 187 mg P, 3.55 mg Zn.

The main beef quality attribute is represented by tenderness which depends on different factors such as chemical, physical, and biochemical ones [5]. Beside this the animal species, age, type of nutrition, gender, and muscle type dictate the tenderness of the meat and also the state of muscular fibres after slaughter [6]. Myofibrillar proteins, connective tissue proteins, and collagen are the main compounds involved in meat tenderness [1]. Meat containing more collagen has been reported as less tender, while high amounts of intramuscular fat could improve this property [5]. Another important characteristic in beef meat is marbling or intramuscular fat which could have a direct impact on the sensory acceptance of it. In this line, Shahrai et al. [7] reported that Longissimus dorsi from Angus beef reached a percentage of 20.87% for marbling, meanwhile, Brahman and Kedah-Kelantan breeds, reached values of 12.17% and 6.86%, respectively, showing once again its superior quality.

Several methods were used so far for improving the meat tenderness, of which marination was considered appropriate because it improved the palatability and the water-holding capacity of meats. The action of the marinating process is based on the decrease of pH by using an alkaline or acidic solution [8]. The protein solubility and the proteases are also processes which occur and improve tenderness [6]. Moreover, the myofibrillar proteins such as actin, myosin, and actomyosin complexes, which are responsible for the water entrapment, increase the meat water-holding capacity (WHC) [9]. Marination process is defined as a treatment of meat with different ingredients such as herbs, spices, organic acids, salt, oil, aiming to tenderise and enhance its flavour [10]. Tenderisation is defined as a process which aims to improve the meat tenderness through chemical, enzymatic treatments or different physical forces [1]. Nowadays consumers are oriented toward natural products which are processed without chemical treatments or additives. Thus, marination with different herbs or oils, but also with enzymes, might be considered as the most suitable procedure for improving meat quality and meeting the consumers expectations. A positive influence on the consumer satisfaction score regarding tenderness was observed to be influenced by marbling, which is defined by Roudbari et al., (2020) as being the intramuscular fat and its dispersion within the lean meat [11].

Oregano, rosemary, and juniper essential oils were used to prepare a marinade along with oil, beer, or lemon and it was concluded that their usage might represent a promising strategy to improve both the qualitative and the sensory characteristics as well as the safety of meat products [12]. These marinades are also imparting novel peculiar sensorial traits to the meats, offering a diversified choice for the consumers. Moreover, plant essential oils are also considered good substituents for the chemical and synthetic antimicrobials and antioxidants substances which are commonly used in meat processing in order to combat the food borne pathogens or spoilage organisms, but also for inhibiting lipid oxidation, thus for prolonging shelf life [13].

The shelf life of meat is almost 24 h at ambient temperature and up to three days at refrigerated temperature (0–7 °C), vacuum packaging of fresh meat being a novel practice well accepted by the population. One of the major drawbacks for this method is the alteration of colour [13], but marination could solve this issue by preserving even more the sensory quality of meat covered in marinade. The addition of spices is also reported to inhibit the formation of highly mutagenic heterocyclic aromatic amines (HAAs), known to possess carcinogenic effects, which occur after the meat is grilled, fried, or broiled at high temperatures [14].

Olive oil is widely accepted as one of the healthiest oils, rich in polyunsaturated fatty acids and antioxidant compounds. Its use has already been explored as an ingredient in marinades for chicken breasts combined with high pressure processing, where improved chemical, health, and sensory qualities were observed [15], but also in a marinade which could inhibit the formation of HAAs in cooked beef patties [14]. Rosemary and its extracts are antifungal, antioxidant and antibacterial ingredients, but their use in foods is limited because of their odour, colour and taste [16]. The rosemary aromatisation of olive oil (extra virgin type) is a smart combination which led to increased total phenolics and carotenoids content and maximised free radical scavenging activity of the mixture [17].

Pumpkin oil is rich in phenolic compounds, valuable minerals, tocopherols, phytosterols and carotenoids, which along with the omega-6 polyunsaturated fatty acid, are imparting to it high nutritional, nutraceutical or therapeutic value [18], thus its usage as an ingredient in meats marinades would increase its overall consumption. Oregano has been added in different forms (fresh, grounded, extract or as essential oil) to different meat products (lamb meat burger, chicken meat patties) in order to increase the antioxidant and antimicrobial activity, but also the quality and stability of the product [19,20].

Sunflower oil consists mainly of 55–65% polyunsaturated linoleic fatty acid, 20–30% oleic monounsaturated fatty acid and the rest are saturated fatty acids [21]. It is used for frying, due to its high smoke point, oxidative stability, tand ability to transfer significant amounts of linoleic fatty acids and tocopherols to fried products. For this reason, and because it is economically reasonable, it should be used as an ingredient in good quality meat marinades. Moreover, walnut oil is an important source for minerals (such as magnesium, iron, phosphorus), antioxidants (vit. C and vit. E) and polyunsaturated fatty acids (PUFA). Walnut oil has proven therapeutic properties in lowering cholesterol, anti-inflammatory, antioxidant and anti-allergenic effects, so it is desired to increase its consumption levels.

Sesame oil was recently used as an ingredient in meatballs, and it inhibited the growth of inoculated foodborne pathogen and exhibited antioxidant effect, improving safety and quality of the products [22]. Sesame oil included in a marinade was also shown to reduce mutagenicity in roasted beef [23].

The aim of the present study was to investigate the potential effect of aromatic plants and cold pressed oils on nutritional, textural and sensorial characteristics of Black Angus beef sirloin meat, stored at 4 °C for 120 h. For this reason, different marinades of olive oil and fresh rosemary, pumpkin oil and fresh oregano, sunflower oil and thyme, walnut oil and fresh basil, sesame oil and ginger were prepared. The antimicrobial and antioxidant activity exhibited towards meat and poultry by rosemary, oregano, thyme and basil, as well as ginger, was recently reviewed by Aziz and Karboune [24].

## 2. Materials and Methods

### 2.1. Standards and Reagents

Organic acid standards were purchased from Fluka (Fluka, Munich, Germany). Luteolin, gallic acid and chlorogenic acid (with a purity > 99%) were purchased from Supelco (Sigma-Aldrich, St. Louis, MO, USA) Analytical reagents and chemicals were achieved from Supelco (Sigma Aldrich St. Louis, MO, USA). All reagents were of analytical grade. MF-MilliporeTM Membrane Filter (0.45 µm) from Merck (Darmstadt, Germany) were used for sample filtration before further analysis and water was previously purified with a Millipore Direct-Q UV system from Merck (Merk, Darmstadt, Germany).

### 2.2. Raw Materials

#### 2.2.1. Marination and Storage of the Samples

The animals were fed ad libitum a total mixed ration based on corn/maize silage (55%), grass-clover silage (15%), corn grains (14%), barley (6%), by-products (10%) and mineral-vitamin salt. The young bulls were sacrificed at the slaughter-house Karpaten Meat SA, from Sibiu, Romania and the sirloin samples were kindly offered for the present study.

The sirloin muscles were marinaded with different fresh seasoning plants and oils as follows: olive oil and rosemary, pumpkin oil and fresh oregano, sunflower oil and thyme, walnut oil and fresh basil, sesame oil and ginger plant, respectively. A control sample spiced with salt and pepper was prepared and stored in the same conditions as the marinated samples (Table 1).

All herbs were purchased freshly from specialised stores from Cluj-Napoca, Romania. The plants were cleaned and washed with tap water and the excess water was drained. Afterwards, they were chopped manually into small pieces and added into the corresponding oil. The cold pressed oils were purchased from Luna Solai, a local company from Luna, Romania.

**Figure 1:**
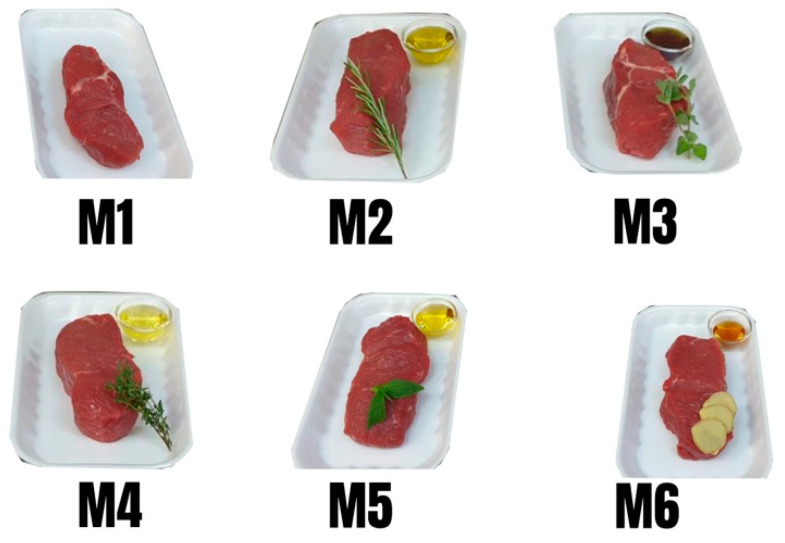
Samples before the marination process.

The meat pieces were cut along the muscular fibres at a final size of (10 × 6 × 4 cm, as showed in Figure 1) with a weight of 370 g and the marination process was made according to Istrati el al. [25] with some modifications and the marinated recipes are presented in the Table 1. Once marinated, the meat samples were stored at 4 °C, for 120 h.

#### 2.2.2. The Cooking Process of the Marinated Beef Samples

The samples were grilled at a temperature of 200 °C until an internal temperature of 71 °C was reached. The temperature was measured using a HACCP digital thermometer (Hendi, Utrecht, The Netherlands) and the samples were grilled for 4 min on each side.

### 2.3. Analysis of the Grilled Marinated Beef Samples

#### 2.3.1. pH-Measurement, Marinade Absorption and Cooking Loss

pH determination was made by using a WTW pH-meter (Hanna Instruments, Vöhringen, Germany) with direct immersion of the glass electrodes in meat sample previously homogenised, as described by Roudbari et al. [10]. Absorption of the marinade was determined according to the method applied by Sengun at al., [9]. Briefly, the percentage of marinade absorption was calculated by the difference between the samples after marination and before the aging process, divided by the weight before marination. The cooking loss was expressed in % and represents the difference between the marinated samples and the cooked ones, as described by Mielnik et al. [26].

#### 2.3.2. Lipids and Fatty Acid Composition

The total lipids (TLs) of the samples (5 g) were extracted using a solvent with chloroform: methanol mixture (2:1, *v/v*), according to the method described by Dulf et al. [27].

Fatty acid methyl esters (FAMEs) of the TLs were prepared by acid-catalysed transesterification using 1% sulfuric acid in methanol [28]. The FAMEs were determined with a gas chromatograph (GC) coupled to a mass spectrometer (MS) (PerkinElmer, Inc., Shelton, CT, USA) as described by [29]. Briefly, 0.5 μL sample was injected into a 60 m × 0.25 mm i.d., 0.25 μm film thickness Supelcowax 10 capillary columns (Supelco Inc., Bellefonte, PA, USA). The operation conditions are as follows: injector temperature 210 °C; helium carrier gas flow rate 0.8 mL/min; split ratio 1:24; oven temperature 140 °C (hold 5 min) to 240 °C at 4 °C/min (hold 30 min); electron impact ionisation voltage 70 eV; trap current 100 μA; ion source temperature 150 °C; mass range 22−395 *m*/*z* (0.14 scans/s with an intermediate time of 0.02 s between the scans).

The identification of FAMEs was accomplished by comparing their retention times with those of known standards (37 components FAME Mix, Supelco no. 47885-U) and the resulting mass spectra to those in the database (NIST MS Search 2.0). The amount of each fatty acid was expressed as peak area percentage of total fatty acids.

#### 2.3.3. Phenolic Compounds Determination Using HPLC/DAD/ESI-MS

Ultrasound assisted extraction (UAE) was used for the extraction of the samples, as described by Călinoiu and Vodnar [30]. Briefly, 5 g of sample was mixed with 10 mL of methanol and 1% HCl, vortexed for 1 min, sonicated for 60 min in a heated ultrasonic bath Elmasonic E 15H (Elma Schmidbauer, GmnH, Singen, Germany) and centrifugated at 2300× *g* for 10 min with an Eppendorf 5804 centrifuge (Eppendorf, Hamburg, Germany) at 24 °C. The supernatant was filtered through a 0.45-µm nylon filter (Millipore, Merk KGaA, Darmstadt, Germany) and 20 µL was injected into the HLPC system (Agilent Technologies 1200 Series, Japan, Kyoto), as previously described by Igual et al. [31]. HPLC system was equipped with a pump, autosampler, DAD detector, and MS-6110 single quadrupole API-electrospray detector.

For the separation of the compounds, an XDB C18 Eclipse column (4.5 × 150 mm, 5 mm) (Agilent Technologies Inc., Santa Clara, CA, USA) was used, having mobile phase (A) made of water acidified by acetic acid 0.1% (*v/v*) and phrase B composed of acetonitrile acidified by acetic acid 0.1% (99:1, *v/v*) with a flow rate of 0.5 mL/min. Mass spectrometric detection on ESI positive mode was used on the following parameters: capillary voltage: 3000 V, temperature 350 °C, *m*/*z*:120–1200, full-scan, nitrogen flow 7 L/min. Agilent ChemStation software Rev B.04.02 SP1 (Agilent Technologies Inc., Palo Alto, California, CA, USA) was used for acquiring results interpretation. The amount of flavonoids was quantified using luteolin calibration curve (r^2^ = 0.9972); meanwhile, for hydroxybenzoic acids gallic acid curve was used (r^2^ = 0.9978). The hydroxycinnamic acids were quantified as chlorogenic acid (r^2^ = 0.9937).

#### 2.3.4. Organic Acid Determination through HPLC-RID

Lactic, malic, oxalic, citric and tartaric acids were analysed by high-performance liquid chromatography (Agilent Technologies Inc., Kyoto, Japan) coupled with refractive index detector (Agilent Technologies Inc., Santa Clara, CA, USA), as described by Chiș et al. [32]. Briefly, 5 g of sample was mixed with 10 mL of ultrapure water, vortexed for 1 min and sonicated for 60 min in a heated ultrasonic bath Elmasonic E 15H (Elma Schmidbauer GmnH, Singen, Germany). Afterward, the sample was centrifugated at 2300× *g* for 10 min with an Eppendorf 5804 centrifuge (Hamburg, Germany), filtered and 20µL was injected in the HLPC system.

A Polaris Hi-Plex H, 300 × 7.7 mm column (Agilent Technologies Inc., Santa Clara, CA, USA) was used for the compound separation with 5 mM H_2_SO_4_ mobile phase at a flow rate of 0.6 mL/min. The column temperature was 80 °C, RID temperature was 35 °C and the compounds elution was made for 25 min. OpenLab software-ChemStation (Agilent Technologies Inc., Santa Clara, CA, USA) was used for data acquisition and interpretation of the results. The retention times of the organic acids were: 13.68, 10.83, 10.01, 9.34, 7.86 min for lactic, malic, tartaric, citric and oxalic acids, respectively.

#### 2.3.5. Texture Profile Analysis

The texture profile analysis was evaluated by using CT 3 Texture Analyser with Texture Pro CT V1.6 soft-ware (Brookfield Engineering Laboratories Inc., Middleboro, MA, USA), according to the method described by Su et al. [33] and Ayyash et al. [34].

The CT 3 Texture Analyser was equipped with a 10 kg load cell and the TA44 Brookfield Kit Probe (4 mm Diameter, Stainless Steel 10 g). The samples were subjected to a double compression, as follows: 50% target deformation, 1 mm s^−1^ test and post-test speed, 5 g trigger load, and 5 s recovery time.

Each piece of meat was cut manually at 30 × 30 × 30 mm (l × w × h) approximately perpendicular to the muscle fibre direction and analysed immediately after preparation.

#### 2.3.6. Sensory Analysis of the Cooked Marinated Samples

Sensory evaluations of the cooked samples were performed using hedonic test according to the method described by Yeh et al. [35] with slight modifications. The sensory characteristics such as appearance, colour, aroma, tenderness, juiciness, taste and flavour and overall acceptability were analysed by 30 voluntary panellists (60% female and 40% male) with ages between 24 and 62 years old (mean value 39.16 ± 9.21) and with experience in cooking beef between 1 year (3 panellists) and more than 10 years (5 panellists) and 22 panellists with experience between this time frame. Informed consent was obtained from all subjects involved in the study. Ethical review and approval were waived for this study because the panellists were healthy, and the study did not employ any nutritional intervention. Samples were safe from the microbiological point of view (tests were performed before the sensory analysis, data not shown). The collected personal data respect confidentiality in accordance with the requirements of Regulation (EU) 2016/679 [36] on the protection of individuals with regard to the processing of personal data and free circulation (GDPR) and are used only for scientific purposes. The statistical processing of the data provided was analysed at the sample level and does not present the individual responses in any scientific publication. The obtained information was used only by the research team with the purpose of this scientific publication.

The analysis was performed in a restaurant, to maintain the safe distance between the panellists and in close proximity to the kitchen with professional cooking devices in order to assure the same quality of preparation of each beef sample. Each panellist evaluated two replications of each sample. Six sensory characteristics were rated on the nine-point hedonic scale: colour, aroma, tenderness, juiciness, taste and flavour, overall appreciation (1 = dislike extremely, 9 = like extremely).

The panellists were minimally trained with the hedonic scale (1 session × 2 h) and the sensory attributes. Tenderness was evaluated as the easiness to chew the beef sample, while juiciness was evaluated as the quantity of saliva absorbed by the beef sample. Both tenderness and juiciness were recorded as subjective perceptions on hedonic scale. The quality of consumption was evaluated also using the following scale: unsatisfactory, good everyday quality, better than everyday quality and premium quality according to [37].

#### 2.3.7. Statistical Analysis

Duncan multiple comparison test by performing SPSS version 19 software (IBM Corp., Armonk, NY, USA) was used for data analysis. The results are expressed as means ± standard deviations of three independent (*n* = 3) assays. Minitab 19.1 (Minitab Inc., State College, PA, USA) at 95 confidence level was used to calculate Pearson correlation.

## 3. Results and Discussion

### 3.1. Results of Analysis Obtained for Oils, Plants Herbs Used for Preparation of Marinades and Marinated Beef Samples

#### 3.1.1. pH and Marinade Absorption Values

With respect to pH samples (Table 2) the control marinated sample reached a final value of 5.2, meanwhile, the M_2_ sample reached the lowest value of 4.9. The initial pH values of oils and plants mixtures are as follows: M_1_ (5.45), M_2_ (4.66), M_3_ (4.95), M_4_ (5.00), M_5_ (4.91), M_6_ (4.82). The changes of pH values during marination could be influenced by the marinade formulation, type of meat and duration, as demonstrated by Roubdari et al. [10]. The M_2_ lowest value could be justified by the lowest pH of initial marinade formulation, but also by a long time of application of the marinade. Removing the meat pH value away from the isoelectric point of red meat (5.2–5.3) will increase the water binding of muscle proteins, enhancing the water structure amount. In this line, [38] showed that the pH values of chicken meat decrease significantly with the addition of the acetic acid, improving the meat water-holding capacity. This idea is supported also by Gómez et al. [39] who showed that during beef marination, the pH decreases from 6.23 to 4.67. The marinade absorption values were in the range of 1.25% to 3.89%. The values increased with increasing the aging time. This is in line with Sengun et al. [9] who reported marinade absorption values for beef samples between 2.99 and 4.01%.

#### 3.1.2. Fatty Acids and Volatile Profile Composition of Oils Used for Aging Meat

The PUFA/SFA index which offers a hint over the impact of the fat containing foods on consumers cardiovascular health (CVH) [40], indicates that the walnut oil with a ratio of 9.74 and the sunflower oil with a ratio of 7.30 are highly recommended to be included in diets.

It is well-known that oils rich in polyunsaturated fatty acids are more prone to degradation and oxidation due to light, oxygen or temperature, thus their application as marination medium might affect the meat quality during storage or cooking. The free radicals and metabolites generated from PUFA oxidation may also result in off-flavours. Linoleic acid is predominant in the composition of the sunflower oil (66.40%) and walnut oil (65.58%), but it is also present in pumpkin seed oil (46.09%) or sesame seed oil (46.94%), as highlighted in Table 3. Hexanal, heptanal and octenal and their derivates are the major volatile oxidation compound of this fatty acids, yet it was not detected in the volatile compounds analysis for the samples maturated or cooked.

Olive oil is abundant in oleic acid, a monounsaturated fatty acid which along with vaccenic acid and palmitoleic also offer nutritional value to the product but make it more prone to oxidation and decompose to short-chain alkanes, aldehydes and alcohols.

Besides their nutritional impact, fats might have a positive role in the texture of the final products and might be able to crystallise under external conditions, allowing a better water-holding capacity of meat during marination. Fat can be affected by external crystallisation conditions such as cooling rate and storage temperature or storage time, in terms of particle sizes or polymorphism. Fat crystallisation include three steps, as follows: nucleation, the first stage of crystallisation, the secondary nucleation when new nuclei are created as a result of inhomogeneous growth on primary crystals forming larger structures, and the third phase called crystal growth [41]. The formation of fat crystals during meat marination is a possible explanation that can explain the fact that oils improve the meat texture.

#### 3.1.3. Polyphenols of the Herbs, Oils and Marinated Beef Samples

Table 4 displays the total amounts of phenolic compounds identified in rosemary (6.01 mg/g), thyme (4.25 mg/g), oregano (3.79 mg/g), basil (3.44 mg/g) and ginger (1.25 mg/g). Phenolic compounds present a co-antioxidant or antioxidant capacity and they act as radical scavengers of the lipid peroxidation chain reactions or deactivate the active species that are precursors of free radicals [42].

The potential of using naturally occurring polyphenols in the control of meat products oxidative state or antimicrobial barrier has been recently reviewed for fresh meat, and also for meat preparations, hence concluding that due to numerous benefits which they exhibit, it is likely for them to become innovative tools integrated into meat systems; however, further research is needed in order to evaluate how other preservation methods such as vacuum packaging interfere in their action [43,44].

Oregano (*Origanum vulgare*), rosemary (*Rosmarinus officinalis*), thyme (*Thymus vulgaris*) are the most frequently used herbs and spices for meat and meat products together with basil and ginger [43]. The biggest amount of phenolic compound was identified in rosemary plant with an amount of 6.78 mg/g, meanwhile the lowest amount was reached by ginger (1.25 mg/g), as illustrated in Table 4.

Table 5 displays the total amounts of phenolic compounds identified in oils used for preparing the marinades, of which olive oil was the most abundant in polyphenolic compounds with a total amount detected of 154.16 μg/g, followed by sunflower oil (16.88 μg/g), pumpkin seed oil (14.38 μg/g) and walnut oil (9.35 μg/g), respectively.

The phenolic compounds can be divided into eight main groups, as follows: flavones, hydroxycinnamic acids, phenolic terpenes, phenolic acids, tyrosols, lignans, hydroxyphenylpropenes and hydroxybenzaldehyde.

The use of herbs in marinated samples influence in a positive way the phenolic content of meat samples, leading to samples enriched in phenols. The individual phenolic compounds determined in the herbs and plants added as ingredients to the marinades are shown in Table 4. Luteolin-gluconoride rosmarinic acid (3.12 mg/g), nepertin (0.64 mg/g), carnosic acid (0.62 mg/g) and cirsimarin (0.43 mg/g) were the main compounds from rosemary. Thyme contained a higher concentration in Luteolin-gluconoride rosemarinic acid (1.87 mg/g), carnasol (0.62 mg/g), rosemarinic acid (0.14 mg/g) and homoplantagin (0.37 mg/g).) Basil was rich in chicoric acid 2.31 (mg/g), a compound which is specific for this herb, and in rosemarinic acid (0.55 mg/g), caftaric acid (0.32 mg/g) and carnosol (0.19 mg/g). Specific polyphenols were also determined in ginger, from the hydroxyphenylpropene class namely gingerol 0.81 mg/g, shogaol 0.29 mg/g and paradol 0.13 mg/g.

With respect to cold pressed oils phenolic compounds, from flavones group, gallocatechin reached the highest amount in walnut oil (1.53 μg/g), from hydroxycinnamic acids group dicaffeoylquinic acid 1 reached a value of 3.63 μg/g, hydroxybenzoic acids were mainly represented by syringic acid (4.38 μg/g) and ellagic acid (1.99 μg/g); from tyrosols group, oleoropein from olive oil registered the highest amount (43.79 μg/g), isolariciresinol amount (30,22 μg/g) represent the main compound from lignans group, meanwhile vanillin (2.63 μg/g) and juglona (1.23 μg/g) represent hydroxybenzaldehide and naphtoquinone groups, as showed in Table 5.

Regarding the phenolic compounds identified in marinated meat (data available in Supplementary Materials) the main flavones compound from M_2_24h was represented by luteolin-gluconoride rosemarinic acid (62.04 μg/g) and cirsimaritin (59.61 μg/g); hydroxybenzoic acid (205.82 μg/g) was the main compound from phenolic acid group, meanwhile, oleoropein derivate (43.79 μg/g) was the main compound from tyrosol group. The polyphenols from olive oil used in marinade preparation could affect microbial inhibition through the selective inhibition of microbial ATP synthase, leading to low microbes cellular energy resulting in bacterial cell death [44]. With respect to M_3_24h the main identified compounds were represented by hydroxybenzoic acid (201.06 μg/g), followed by dihydroxybenzoic acid (99.17 μg/g), carnosol (39.99 μg/g) and luteolin-glucuronide (30.93 μg/g).

Hydroxytyrosol, a phenolic derivate and oleoropein, a phenolic compound from the secoiridoids class, are reported by literature as minor components of olive oil which present biological properties and prevent gestational diabetes mellitus [45]. In the olive oil involved in the marinaded formulation was detected a total amount of 30.22 (μg/g). This compound was also detected in meats. In M_2_24h hydroxytyrosol has a concentration of 9.71 μg/g and its concentration is slightly decreased to 9.01 μg/g (72 h) and 8.40 μg/g (120 h). In the thermally treated meat, the phenolic compound is absent, thus it can be affirmed that it is a thermolabile compound. In M_2_24h, oleoropein was detected in 43.79 and is decreased to 39.28 (72 h) and 38.83 (120 h) and the same amount and decreasing trend was also registered for cooked meat.

Hydroxybenzoic acid (248.59 μg/g), dihydroxybenzoic acid (41.18μg/g), dicaffeoylquinic acid (33.13 μg/g) and luteolin-glucuronide (30.05 μg/g) were the main phenolic compounds identified in M424h, while hydroxybenzoic acid (237.88 μg/g), dihydroxybenzoic acid (71.33 μg/g), carnasol (30.89 μg/g) and rosmarinic acid (27.73 μg/g) were the main phenolic compounds identified in M_5_24h.

Regarding the M_6_24h and M_5_24h, hydroxybenzoic and dihydroxybenzoic acids were the main compounds, but paradol (36.83 μg/g), sesamin (27.91 μg/g) and gingerol (23.15 μg/g) were identified only in M_6_M24h sample, due to the chemical composition of ginger. Sesamol favourably delayed the lipid oxidation of meatballs and exhibited antioxidant activities and could be used as a natural additive in order to improve the microbial quality and therefore, improving the shelf life of meatballs [23]. It was also mentioned that sesamin could be involved in the lipid oxidation systems, leading to an enhancement of the vitamin E antioxidant activity [43].

The natural phenolic compounds such as phenolic acids with an aromatic ring from which the main groups are hydroxybenzoic acids, hydroxycinnamic acid and flavonoids are claimed by the literature as having antioxidant, antifungal, antimicrobial and antiviral functions [41]. The hydroxybenzoic acids, as reported by Murkovic, [46] derived directly from benzoic acid and represent a complex structure between lignin’s and hydrolysable tannins.

#### 3.1.4. Organic Acid Marinated Samples

Organic acids are GRAS (Generally Recognised as Safe), low cost and are claimed to have minor influence on the products sensory changes [47]. Regarding their technological functionality, lactic and acetic acids are considered to be preservatives, meanwhile, malic, citric and tartaric are considered acidifiers [48]. Organic acids could affect in a positive way the meat texture during marination. It was previously proved that thickness and fibre diameter values decreased and collagen fibre were disordered through marination with weak organic acids from fruit vinegars [10].

The presence of malic, oxalic and citric acid in meat marinated samples, as presented in Table 6 could be justified by their presence in oils and plants used for marination. For instance, olive fruits are claimed to have an increased content of organic acids (oxalic, citric, malic and succinic) in the last maturation months, as highlighted by Nergiz et al. [49]. The presence of malic acid in walnut oil was highlighted by Radu et al. [50] and citric and malic acids were identified in pumpkin species [51]. Citric acid is used in beef muscle marination mainly because of its positive influence on tenderness and WHC, being considered as a food acidulant [52]. Moreover, it seems that citric acid could be involved in the inhibition of meat lipid oxidation [53]. Lactic and citric acids could have a main role in preservation of the meat products through their antimicrobial activity against microflora identified in meat. Decontamination of red meat carcasses with lactic acid in a range of 1% to 2% could be performed with a minimum effect on the sensory quality of meat [54]. Organic acids have the ability to act as permeabilizers to pass the outer membrane of bacteria such as Gram negative bacteria, enabling the entrance of other hydrophobic molecules [55].

In the present study, the biggest amount of tartaric acid was identified in meat control sample (M_1_24h), registering a value of 1847.19 μg/g and decreasing its value through marination at a final value of 1100.03 μg/g after 120 h of marination. With respect to M_2_ sample, lactic and citric acid increased its value through maturation time, meanwhile, tartaric acid decreased its value, as presented in Table 6. Citric, lactic and acetic acids are considered by the European food legislation as being quantum satis, with no maximum level addition in food industry [48].

Mani-López et al. [47] showed that organic acids are strictly correlated with pH value and their antimicrobial activity is enhanced as the pH amount decreases. Adamczak et al. [56] showed that citric and malic acids identified in plants could have antibacterial activity against Gram positive and Gram negative bacteria. It seems that acidic marinades lead to the weakening of meat structures due to the pH decrease. During cooking, collagen is converted into gelatin, increasing the meat tenderness [54].

#### 3.1.5. Textural Properties of Marinated Sirloins

The textural analysed parameters (hardness, resilience, cohesiveness, gumminess and chewiness) are listed in Table 7. The peak forces of the first and second compression cycles are defined as hardness 1 and hardness 2, meanwhile the internal resistance of food structure and the extension level to which the product could be deformed is expressed through cohesiveness. Gumminess is a parameter strictly correlated with hardness and cohesiveness [57].

One of the main problems in meat industry was claimed to be the inconsistency of beef tenderness caused by the differences in myofibrillar and connective tissue proteins [24]. Marination had a positive effect on all analysed samples parameters, resulting in a reduction of sirloin hardness, chewiness and gumminess and positive effect on resilience and cohesiveness. The improvement of the aforementioned parameters through marination process was also highlighted by Istrati et al. [25]. A possible explanation of the parameters improvement could be that during marination process, the organic acids enhanced the muscle structure decay and the collagen connective tissue could be broke [24]. Furthermore, because of the marinades ionic characteristics of salt, the space among the protein molecules is partially unfolded or opened enhancing the water-binding sites availability leading to a highly concentrated protein network [9]. Malic and citric acid could be used in the optimisation of the red meat textural parameters, as previously showed by Botinestean et al. [58]. Briefly, they demonstrated that mainly citric acid could have positive effects on red meat texture parameters, leading to a meat that could be consumed also by older consumers. It seems that citric acid decreased the hardness meat value through protein meat partial denaturation during cooking. The organic acids meat softening effects was also mentioned by Chang et al. [59] through improving the connective meat tissue collagen property and textural characteristics.

### 3.2. pH, Water Loss, Phenolic Compounds, Organic Acid, Texture and Sensory Analysis of the Processed Sirloin Samples

#### 3.2.1. pH Value and Water Loss

Gómez et al. [39], showed that pH of beef during cooking increased until 6.17. This could be explained by the calcium and magnesium protein releasing ions, reducing the available carboxylic proteins group through thermal treatment. Regarding the cooked meat samples, the pH increased compared to the marinated samples. For instance, the M_2_120h sample registered a pH value of 4.9, meanwhile, the M_2_T120h reached a pH value of 5.27, as presented in Table 8.

With respect to the water loss samples during cooking (presented in Table 8) the smallest loss during thermal treatment was registered in sample M_2_120h (19.10%). This could be justified by the decrease of pH during marination, which improves the meat water-holding capacity [8,37]. Furthermore, a main role in improving the water-holding capacity of beef meat is of citric acid, a food acidulant [60] which was identified in all analysed samples. Marinades are responsible also for the reducing water loss during cooking owing to the developing of an extra succulent texture on the meat surface [61].

#### 3.2.2. Treated Marinated Meat Phenolic Acid Samples Results

Phenolic acid compounds have a main role in the formation of polycyclic aromatic hydrocarbons (PAF), a class of organic compounds composed of fused aromatic rings with a negative influence on the human bod. According to Wang et al. [62] the exposure to PAF could enhance the formation of different types of cancer such as breast, lung or stomach cancers. Phenolic compounds from marinades such as gallic acid, ferulic or protocatechuic acid exhibited highly inhibitory effects on charcoal-grilled chicken wings PAF formation.

With respect to the quantity of total phenolic acid content, the amount decreased compared to the marinated samples, probably due to the thermal treatment, which influence in a negative way the total amount of phenolic acids. For example, for sample M_2_120h the total identified compounds were 674.80 μg/g, meanwhile the total amount for the thermal treated samples was 585.38 μg/g. Generally, the flavones, hydroxycinnamic acids, tyrosol, lignans and hydroxyphenylpropene groups decreased their amount through thermal treatment, except hydroxybenzoic acid, a compound from phenolic acid group, as presented in Supplementary Materials. Hydroxybenzoic acid derivates are considered phenolic metabolites and are identified in conjugates forms; as mentioned by Tomás-Barberán et al. [63], processing could have a positive effect in their bioavailability, increasing its value. Therefore, a possible explanation of the increased hydroxybenzoic acid amount during thermal treatment of meat, could be the fact of increasing its bioavailability. High Pearson’s values (0.985, 0.961, 0.808, 0.998, 0.959, 0.993) highlighted a strong relationship between the phenolic content of M_1_, M_2_, M_3_, M_4_, M_5_, M_6_ marinated samples and their treated samples.

#### 3.2.3. Organic Acids Content in Treated Meat

Organic acids content of cooked samples is presented in Table 9. The lowest lactic acid content was identified in control sample marinated for 120 h (2255.24 μg/g), meanwhile, the highest level was registered by M_2_T120h (cooked sample marinated with olive oil and rosemary). Lactic acid is involved in the food taste and preservation and could be produced by lactic acid bacteria. For instance, Da Costa et al. [64] showed that different strain of lactic acid bacteria have been isolated from red meat, such as: Lactobacillus sakei and Lactobacillus curvatus. Moreover, the plants used for marination could have also lactic acid bacteria, which could lead to the enrichment of organic acids through marination process. Furthermore, organic acids could influence the texture of the products. As shown by Berge et al. [65], injecting lactic acid in beef muscles caused a decrease of the beef muscle pH to around 4.9 and showed a reduction of collagen toughness, hence improving the meat tenderness through weakening the myofibrillar structure and the connective tissue.

Tartaric, citric and malic acids are considered week organic acids and are widely used in meat marination. For instance, citric acid together with 2% NaCl could be used in marinated beef meat increasing the mechanical strength of connective tissue, decreasing thickness and disordering the collagenous fibres [58]. Furthermore, Braïek et al. [48] showed that due to the addition of 1% acetic acid and 1% lactic acid the sensory quality of treated beef meat was improved in colour, flavour and texture.

#### 3.2.4. Textural Characteristics of Cooked Meats

Consumer satisfaction with the tenderness of a red meat is mainly based on the relationship between mouthfeel and textural properties which include chewiness, hardness, firmness [66]. From the consumers point of view, tenderness is the most important meat palatability trait [25]. Tenderisation is defined as a process which aims to improve the meat tenderness through chemical, enzymatic treatments or different physical forces [1]. During cooking, as a consequence of heating, the meat products will lose a part of the added marinade/water but due to the formation of the gel protein matrix, the water will be retained better [10]. Moreover, as highlighted by Yusop et al. [10] due to the marination process the water loss is minimised by the earlier absorbed marinade and the aforementioned gel could act as a protective barrier against water losses leading to an improvement in tenderness, flavour and moisture in cooked meat. It is also important to mention that salt is involved in the polypeptide chain rigidity loss [58], and also thermal treatment results in decreased values of hardness, gumminess and chewiness [67] through the denaturation of myofibrillar proteins and connective tissue.

In the present study, M_2_T120h registered the lowest hardness, chewiness and gumminess index values, meanwhile resilience and cohesiveness highlighted the biggest values, being statistically different compared to all the samples at the same marination time, as presented in Table 10. Moreover, M_3_T120h and M_5_T120h showed a significant decrease in hardness, gumminess and chewiness during the thermal treatment time, starting from values of 1255, 841, 76.1 and 1246, 1017 and 109.9 at 24 h marination time and reaching values for 120 h marination time of 679, 348 and 50.4 and 583, 422 and 53.4 respectively. Strong positive Pearson’s correlations (0.92, 0.90, 0.91, 0.88, 0.99) were identified between M_2_, M_3_, M_4_, M_5_, M_6_ at 24 h, 72 h, 120 h marinated samples and the cooked ones, from the hardness point of view, emphasising once again that marination has a strong influence on the texture of the final grilled meat. Furthermore, hardness, chewiness, gumminess values decreased with increasing marination time from 24 h to 120 h (as showed in Table 10), meanwhile, resilience, cohesiveness and chewiness decreased their values. The control sample presented a final higher value for hardness, chewiness and gumminess and also a smaller decrease in resilience, cohesiveness and gumminess. This is in line with Istrati et al. [25] who showed that marination of fresh beef slices improved its textural characteristics, mainly on the samples with marinated ingredients. The control sample marinated for 120 h registered a final hardness value of 993 [N], meanwhile the lowest hardness value was registered by M_2_T120h (392 [N]). With respect to gumminess and chewiness, the lowest values were registered by M_2_T120h of 257 [N] and 44 [N], followed by M_3_T120h with values of 348 [N] and 50.40 [N], respectively.

#### 3.2.5. Sensory Analysis

In the present study, the lowest hedonic score was registered by the control sample marinated at 24 h (M_1_24h) with an overall appreciation of 6.57 (as presented in Table 11), meanwhile the highest hedonic score was registered by M_2_120h, with a hedonic score of 8.93. Generally, the values of each sensorial characteristic improved during marination, as presented in Table 11.

Interesting results were obtained when instrumental analysis of texture (hardness, gumminess and chewiness) was compared to hedonic evaluation scores for tenderness, juiciness (subjective perception of the amount of saliva absorbed by the sample) and taste and flavour. Hardness cycle 2 (instrumental) correlated moderately negatively with hedonic evaluation of tenderness (Pearson value −0.63, *p* < 0.05) and juiciness (Pearson value −0.61, *p* < 0.05). However, hardness cycle 1 (instrumental) strongly correlated with hedonic evaluation of tenderness (Pearson value −0.89, *p* < 0.001) and taste and flavour (Pearson value −0.87, *p* < 0.001), but did not correlate with juiciness (Pearson value 0.12, *p* < 0.001).

The improvement of meat quality through marination was sustained also by [68] who showed that meat marination process could enhance the meat sensory properties through increased scores values of the juiciness, flavour and tenderness. The tenderness is closely corelated with the drop of the pH near to the meat isoelectric point (approximately 5.2). Furthermore, the sensation of red meat product juiciness upon the mastication is strictly corelated with the water content of the meat product and saliva released by salivary glands [66].

A strong negative correlation was noticed between the instrumental evaluation of gumminess and hedonic evaluation of tenderness (Pearson value −0.85, *p* < 0.001), juiciness (Pearson value −0.85, *p* < 0.001) and finally, taste and flavour (Pearson value −0.82, *p* < 0.001). The same pattern of strong negative correlation was recorded between the instrumental analysis of chewiness and hedonic evaluation of tenderness (Pearson value −0.78, *p* < 0.001), juiciness (Pearson value −0.77, *p* < 0.001) and taste and flavour, respectively (Pearson value −0.88, *p* < 0.001). Moderate negative correlation was found between instrumental evaluation of resilience and hedonic evaluation of tenderness (Pearson value −0.59, *p* < 0.001) and moderate positive correlation was found between instrumental evaluation of cohesiveness and hedonic evaluation of tenderness (Pearson value 0.41, *p* < 0.001).

With respect to the analysed taste and flavour characteristics, the lowest value was registered by M_1_24h and the highest by M_2_120h, as shown in Table 11. Flavour is a sensorial property defined by [67] as a combination between smell (aroma) and consumer taste responses upon eating red meat. It could be quantified through different laboratory-based methods such as fatty acid profile, pH and mineral content. Several studies have reported that the fatty acid profiles are involved in flavour differences between meat samples. For instance, it had been shown that elevated amount of linolenic acid was reported to have a ‘fishy’ or ‘grassy’ flavour, due to the presence of phyt−2-ene [69]. This could justify the consumer preference through samples marinated in olive oil, considering that it has a significant small content (0.37%) in linolenic acid, compared to walnut oil (8.90%) or even sesame oil (0.48%). Furthermore, it seems that SFAs and MUFAs could emphasise a positive role in Longissimus thoracis young bulls meat flavour, tenderness and juiciness as highlighted by Listrat et al. [70].

On the other hand, the phenolic compounds from plants, could be also involved in the final flavour of foods through their own taste and/or by retarding the formation of other flavour compounds, such as oxidation products [71]. It is also important to mention that thermal degradation of lipids chiefly plays a main role in Maillard reaction. For instance, hexanal, a dominant aldehyde in meat could be produced from the oxidation of linoleic and arachidonic acids [71].

All samples recorded an improvement in the perception of the consumption quality with increasing the marination time. After the first 24 h of marination, all samples recording less than 20% were evaluated as premium quality. However, after 120 h of marination, all samples recording above 40% were evaluated as premium quality, except for the control sample M_1_T120h which had only 33%. These results prove that the aromatics of herbs and oils improved the perception of quality of the samples after marination.

## 4. Conclusions

Beef consumption highly increased in the last 20 years and the trend seems to continue further. This study focused mainly on the effect of different aromatic herbs (namely rosemary, oregano, thyme, basil, ginger) and cold pressed oils (olive oil, pumpkin oil, sunflower oil, walnut oil, sesame oil) on the marination of Black Angus beef meat. The selected aromatic herbs are commonly used either in the European or in the Asian cuisine, therefore, well-known by consumers, however, their use in marinating beef meat is new. Cold pressed oils are very rich in aroma, taste and flavour due to their unique phenolic and aromatic profile. Phenolic acids such as carnosic acid, carnasol, cirsimaritin, rosemarinic acid were identified in cooked meat sample marinated with olive oil and rosemary, meanwhile chlorogenic acid, homoplantaginin (hispidulin-glucoside) tyrosol, chicoric acid, sesamin, paradol and gingerol were mainly identified in samples marinated with pumpkin and oregano, sunflower oil and thyme, walnut oil and basil and sesame oil with ginger, respectively.

As the results of this study, both aromatic herbs and cold pressed oils showed to enrich the meat with aroma and flavour, and positively influence the texture of the beef (mainly tenderness and juiciness) especially after longer marination time. These results were determined by means of instrumental analysis of texture, quantitative evaluation of lipids, flavonoids and polyphenols, organic acids, and confirmed by sensory evaluation with highly experienced panellists. Each aromatic plant and oil used for the purpose of marination presented the characteristic polyphenolic profile, and their influence on the composition and sensory attributes of the marinated samples was significant at 72 h and 120 h of marination compared to the initial moment of evaluation. From the textural point of view, positive strong Pearson’s correlations (above 0.88) were calculated between the hardness of the marinated samples and the grilled ones, emphasising once again the influence of the marination on textural parameters. Tenderness, taste and flavour, juiciness and colour reached highest hedonic scores on sample marinated with olive oil and rosemary. Except for colour which recorded a decrease in terms of hedonic appreciation, all other sensory characteristics improved with marination time and in the presence of the marinade compared to the control (which was seasoned just with salt and powder black pepper).

These are encouraging results which can inspire all actors in the food industry chain (producers, distributers, chefs and consumers) to test and develop various marinades using herbs and oils, therefore, positively influencing the nutritional, textural and sensory attributes of grilled beef sirloin.

## Figures and Tables

**Table 1:** The codification of samples used in the study.

Spices, Herbs and Oils	Duration of Marination		
	24 h	72 h	120 h
	Codes for Marinades		
Salt * and Black pepper **	M_1_24h	M_1_72h	M_1_120h
Rosemary *** and Olive oil ****	M_2_24h	M_2_72h	M_2_120h
Oregano *** and Pumpkin oil ****	M_3_24h	M_3_72h	M_3_120h
Thyme *** and Sunflower oil ****	M_4_24h	M_4_72h	M_4_120h
Basil *** and Walnut oil ****	M_5_24h	M_5_72h	M_5_120h
Ginger *** and Sesame oil ****	M_6_24h	M_6_72h	M_6_120h
	Codes for marinated beef samples		
Salt * and Black pepper **	M_1_M24h	M_1_M72h	M_1_M120h
Rosemary *** and Olive oil ****	M_2_M24h	M_2_M72h	M_2_M120h
Oregano *** and Pumpkin oil ****	M_3_M24h	M_3_M72h	M_3_M120h
Thyme *** and Sunflower oil ****	M_4_M24h	M_4_M72h	M_4_M120h
Basil *** and Walnut oil ****	M_5_M24h	M_5_M72h	M_5_M120h
Ginger *** and Sesame oil ****	M_6_M24h	M_6_M72h	M_6_M120h
	Codes for marinated grilled beef samples		
Salt * and Black pepper **	M_1_T24h	M_1_T72h	M_1_T120h
Rosemary *** and Olive oil ****	M_2_T24h	M_2_T72h	M_2_T120h
Oregano *** and Pumpkin oil ****	M_3_T24h	M_3_T72h	M_3_T120h
Thyme *** and Sunflower oil ****	M_4_T24h	M_4_T72h	M_4_T120h
Basil *** and Walnut oil ****	M_5_T24h	M_5_T72h	M_5_T120h
Ginger *** and Sesame oil ****	M_6_T24h	M_6_T72h	M_6_T120h

* Salt was added as 2% in the marinade mixture. ** Black pepper (ground) was added as 0.50% in the marinade mixture. *** All herbs were added as 0.014 kg/kg from the marinade mixture. **** All oils were added as 0.048 L/kg from the marinade mixture.

**Table 2:** pH and marinade absorption values of the marinated beef samples.

Marinated Meat Samples	pH Value			Marinated Absorption (%)		
Time (h)	24 h	72 h	120 h	24 h	72 h	120 h
M_1_ (control)	5.8 ± 0.02 ^ab,ABC^	5.6 ± 0.01 ^a,BC^	5.5 ± 0.02 ^a,BC^	n.d.	n.d.	n.d.
M_2_	5.5 ± 0.01 ^b,A^	5.2 ± 0.03 ^a,bA^	4.9 ± 0.03 ^a,A^	1.35 ± 0.01 ^a,A^	2.89 ± 0.02 ^b,A^	3.85 ± 0.03 ^c,A^
M_3_	5.9 ± 0.02 ^b,BC^	5.4 ± 0.01 ^a,AB^	5.0 ± 0.01 ^a,A^	1.25 ± 0.05 ^a,A^	2.91 ± 0.03 ^b,A^	3.76 ± 0.01 ^c,A^
M_4_	6.0 ± 0.01 ^b^,^C^	5.8 ± 0.02 ^a,CD^	5.4 ± 0.02 ^a,BC^	1.31 ± 0.04 ^a,A^	2.97 ± 0.01 ^b,A^	3.88 ± 0.02 ^c,A^
M_5_	6.1 ± 0.02 ^b,C^	5.9 ± 0.03 ^b,CD^	5.2 ± 0.03 ^a,AB^	1.27 ± 0.03 ^a,A^	2.93 ± 0.02 ^b,A^	3.84 ± 0.02 ^c,A^
M_6_	5.9 ± 0.03 ^b,BC^	5.3 ± 0.03 ^a,A^	5.0 ± 02 ^a,A^	1.29 ± 0.02 ^a,A^	2.90 ± 0.04 ^b,A^	3.70 ± 0.03 ^c,A^

Mean values of three different determinations followed by standard deviation; M_1_–sirloin marinated only with salt and pepper; M_2_–sirloin marinated with olive oil, rosemary, salt and pepper; M_3_–sirloin marinated with pumpkin oil, oregano, salt and pepper; M_4_–sirloin marinated with sunflower oil, thyme, salt and pepper; M_5_-sirloin marinated with walnut oil, basil, salt and pepper; M_6_-sirloin marinated with sesame oil, ginger, salt and pepper; Values not sharing the same small letter in a row indicate significant difference between the same sample at different moments: 24 h, 72 h, 120 h. Values not sharing the same capital letters indicate the significant difference between samples at the same moment; n.d.—not detected.

**Table 3:** Fatty acids and volatile profile composition of oils used for aging meat.

Shorthand Nomenclature	Fatty Acid Name	Type	Olive Oil (%)	Pumpkin Oil (%)	Sunflower Oil (%)	Walnut Oil (%)	Sesame Oil (%)
16:0	Palmitic	SFA	11.92 ± 0.33 ^d^	12.08 ± 0.45 ^e^	5.95 ± 0.22 ^a^	6.66 ± 0.33 ^ab^	10.00 ± 0.12 ^c^
16:1 (n-7)	Palmitoleic	MUFA	0.93 ± 0.11	n.d.	n.d.	n.d.	n.d.
18:0	Stearic acid	SFA	2.03 ± 0.21 ^ab^	6.02 ± 0.39 ^c^	3.08 ± 0.29 ^b^	0.99 ± 0.03 ^a^	5.48 ± 0.18 ^c^
18:1 (n-9)	Oleic acid	MUFA	75.61 ± 0.15 ^e^	33.22 ± 0.34 ^c^	24.10 ± 0.22 ^b^	17.02 ± 0.18 ^a^	35.31 ± 0.79 ^d^
18:1 (n-7)	Vaccenic acid	MUFA	1.93 ± 0.22 ^d^	2.50 ± 0.33 ^e^	0.40 ± 0.26 ^a^	0.85 ± 0.12 ^ab^	1.79 ± 0.51 ^c^
18:2 (n-6)	Linoleic acid	PUFA	6.96 ± 0.78 ^a^	46.09 ± 1.23 ^b^	66.40 ± 1.02 ^de^	65.58 ± 0.77 ^d^	46.94 ± 1.16 ^b,c^
18:3 (n-3)	Linolenic	PUFA	0.37 ± 0.03	n.d.	n.d.	8.90 ± 0.59	0.48 ± 0.02
20:0	Arachidic acid	SFA	0.24 ± 0.02	n.d.	n.d.	n.d.	n.d.
22:00	Behenic acid	SFA	n.d.	n.d.	0.07 ± 0.01	n.d.	n.d.
Total SFA			14.20 ± 0.56 ^c^	18.09 ± 0.84 ^d^	9.10 ± 0.51 ^b^	7.65 ± 0.36 ^a^	15.48 ± 0.30 ^c^
Total MUFA			78.61 ± 0.48 ^e^	35.82 ± 0.67 ^c^	24.50 ± 0.48 ^b^	17.87 ± 0.30 ^a^	37.10 ± 1.30 ^d^
Total PUFA			7.33 ± 0.81 ^a^	46.09 ± 1.23 ^b^	66.40 ± 1.02 ^d^	74.78 ± 1.36 ^e^	47.40 ± 1.18 ^c^
PUFAs/SFAs			0.52 ^a^	2.55 ^b^	7.30 ^c^	9.74 ^d^	3.06 ^b^

Values not sharing the same small letter in a row indicate significant difference between oils; n.d.—not detected.

**Table 4:** Polyphenolic compounds identified in fresh herbs.

Identified Compound (mg/g)	Rosemary	Thyme	Oregano	Basil	Ginger
Flavones					
Hydroxy-Luteolin-glucoside	0.14 ± 0.02	n.d.	n.d.	n.d.	n.d.
Cirsimaritin	0.18 ± 0.12	n.d.	n.d.	n.d.	n.d.
Nepetrin	0.64 ± 0.22	n.d.	n.d.	n.d.	n.d.
Plantaginin	0.35 ± 0.11	n.d.	n.d.	n.d.	n.d.
Luteolin-acetyl-glucuronide	0.33 ± 0.13 ^b^	0.38 ±0.34 ^c^	0.26 ± 0.11 ^a^	n.d.	n.d.
Cirsimarin	0.43 ± 0.11 ^c^	n.d.	0.13 ± 0.22 ^b^	0.02 ± 0.32 ^a^	n.d.
Luteolin–malonyl–glucoside	n.d.	n.d.	0.52 ± 0.14	n.d.	n.d.
Homoplantaginin	n.d.	0.37 ± 0.13 ^b^	n.d.	0.05 ± 0.11 ^a^	n.d.
Apigenin–glucoside	n.d.	0.28 ± 0.33 ^b^	0.09 ± 0.22 ^a^	n.d.	n.d.
Luteolin–glucoside	n.d.	0.27 ± 0.21 ^b^	0.14 ± 0.22 ^a^	n.d.	n.d.
Hydroxycinnamic acids					
Luteolin-glucuronide Rosemaryic acid	3.12 ± 0.11 ^c^	1.89 ± 0.22 ^b^	1.83 ± 0.27 ^a^	n.d.	n.d.
Rosemaryic acid	n.d.	0.41 ± 0.45 ^a^	0.55 ± 0.56 ^c^	0.52 ± 0.67 ^b^	n.d.
Luteolin-glucuronide Rosemaryic acid	n.d.	n.d.	n.d.	n.d.	n.d.
Caftaric acid	n.d.	n.d.	n.d.	0.32 ± 0.55	n.d.
Chicoric acid	n.d.	n.d.	n.d.	2318.95 ± 0.21	n.d.
Phenolic terpene					
Carnosol	0.15 ± 0.33 ^a^	0.62 ±0.33 ^d^	0.24 ± 0.55 ^c^	0.19 ± 0.23 ^b^	n.d.
Carnosic acid	0.62 ± 0.22	n.d.	n.d.	n.d.	n.d.
Hydroxyphenylpropene					
Paradol	n.d.	n.d.	n.d.	n.d.	0.13 ± 0.44
Gingerol	n.d.	n.d.	n.d.	n.d.	0.81 ± 0.78
Shogaol	n.d.	n.d.	n.d.	n.d.	0.29 ± 0.33
Hydroxybenzaldehide					
Carnosol	0.15 ±0.39 ^a^	0.62 ± 0.44 ^d^	0.24 ± 0.78 ^c^	0.19 ± 0.65 ^b^	n.d.
Carnosic acid	0.62 ± 0.87	n.d.	n.d.	n.d.	n.d.
Total	6.01 ± 2.63 ^e^	4.25 ± 2.45 ^d^	3.79 ± 3.07 ^c^	3.44 ± 2.74 ^b^	1.25 ±1.55 ^a^

Values not sharing the same small letter in a row indicate significant difference between plants; n.d.—not detected.

**Table 5:** Polyphenolic compounds identified in oils.

Identified Compound (μg/g)	Olive Oil	Sunflower Oil	Pumpkin Oil	Walnut Oil	Sesame Oil
Flavones					
Luteolin	1.44 ± 0.45	n.d.	n.d.	n.d.	n.d.
Gallocatechin	n.d.	n.d.	n.d.	1.53 ± 0.34	n.d.
Hydroxycinnamic acids					
Chlorogenic acid	n.d.	1.49 ± 0.48	n.d.	n.d.	n.d.
Dicaffeoylquinic acid 1	n.d.	0.98 ± 0.21 ^a^	3.63 ± 0.29 ^b^	n.d.	n.d.
Dicaffeoylquinic acid 2	n.d.	0.79 ± 0.38	n.d.	n.d.	n.d.
Caftaric acid	n.d.	13.51 ± 0.76	n.d.	n.d.	n.d.
Hydroxybenzoic acids					
Vanillic acid	1.54 ± 0.33	n.d.	n.d.	n.d.	n.d.
Sinapic acid	n.d.	n.d.	n.d.	n.d.	1.65 ± 0.53
Ellagic acid	n.d.	n.d.	n.d.	1.99 ± 0.29	n.d.
Syringic acid	n.d.	n.d.	1.22 ±0.27 ^a^	n.d.	4.38 ± 0.61 ^b^
Tyrosols					
Hydroxytyrosol	30.22 ± 0.65	n.d.	n.d.	n.d.	n.d.
Tyrosol	14.11 ± 0.35	n.d.	n.d.	n.d.	n.d.
Oleoropein	43.79 ± 0.53	n.d.	n.d.	n.d.	n.d.
Oleoropein derivative	15.88 ± 0.30	n.d.	n.d.	n.d.	n.d.
Lignans					
Pinoresinol	2.4 ± 0.54 ^b^	0.11 ± 0.03 ^a^	n.d.	n.d.	n.d.
Acetoxypinoresinol	24.12 ± 0.63 ^c^	n.d.	n.d.	4.6 ± 0.45 ^b^	0.25 ± 0.03 ^a^
Matairesinol	20.66 ± 0.27	n.d.	n.d.	n.d.	n.d.
Isolariciresinol	30.22 ± 0.56 ^b^	n.d.	6.90 ± 0.53 ^a^	n.d.	n.d.
Sesamin	n.d.	n.d.	n.d.	n.d.	10.28 ± 0.72
Sesamolin	n.d.	n.d.	n.d.	n.d.	7.58 ± 0.23
Hydroxybenzaldehide					
Vanilin	n.d.	n.d.	2.63 ± 0.22	n.d.	n.d.
Naphtoquinone					
Juglona	n.d.	n.d.	n.d.	1.23 ± 0.11	n.d.
Total	154.16 ± 4.61 ^e^	16.88 ± 1.86 ^c^	14.38 ± 1.31 ^b^	9.35 ± 1.19 ^a^	24.15 ± 2.12 ^d^

Values not sharing the same small letter in a row indicate significant difference between plants; n.d.—not detected.

**Table 6:** Organic marinated acids content.

Samples	Oxalic (μg/g)	Citric (μg/g)	Tartaric (μg/g)	Malic (μg/g)	Lactic (μg/g)
M_1_24h	21860.49 ± 1.23 ^c,R^	3592.45 ± 0.33 ^c,O^	1847.19 ± 0.78 ^c,P^	859.00 ± 0.47 ^b,R^	6574.54 ± 0.89 ^c,Q^
M_1_72h	13422.09 ± 0.89 ^b,0^	2876.32 ± 0.88 ^b,H^	1503.22 ± 0.75 ^b,N^	829.02 ± 0.39 ^a,P^	5780.93 ± 0.99 ^b,O^
M_1_120h	4367.35 ± 1.03 ^a,B^	2228.82 ± 0.63 ^a,D^	1100.03 ± 0.39 ^a,E^	850.62 ± 0.73 ^b,Q^	4223.17 ± 0.21 ^a,C^
M_2_24h	6456.40 ± 0.62 ^b,H^	3268.91 ± 0.81 ^a,L^	1416.44 ± 0.39 ^b,M^	813.72 ± 0.78 ^b,O^	4517.27 ± 1.30 ^a,F^
M_2_72h	6023.23 ± 0.39 ^a,F^	3512.78 ± 0.78 ^b,M^	1367.90 ± 0.38 ^a,K^	801.78 ± 0.59 ^b,N^	5131.78 ± 1.65 ^b,K^
M_2_120h	5961.24 ± 1.29 ^a,E^	3632.05 ± 0.89 ^c,P^	1343.37 ± 0.55 ^a,J^	783.70 ± 0.44 ^a,M^	5653.69 ± 0.56 ^c,M^
M_3_24h	12621.95 ± 0.78 ^c,N^	3883.12 ± 1.49 ^c,Q^	1382.86 ± 0.82 ^c,L^	608.02 ± 0.78 ^a,G^	6235.59 ± 0.34 ^c,P^
M_3_72h	7234.09 ± 0.55 ^b,J^	3562.22 ± 1.05 ^b,N^	1256.09 ± 1.20 ^b,I^	657.13 ± 0.88 ^b,I^	5731.09 ± 49 ^b,N^
M_3_120h	3576.84 ± 0.89 ^a,A^	2949.46 ± 1.30 ^a,J^	1107.93 ± 1.42 ^a,F^	755.77 ± 0.31 ^c,L^	5245.97 ± 0.31 ^a,L^
M_4_24h	4583.12 ± 0.93 ^a,C^	2436.40 ± 0.93 ^c,E^	681.80 ± 1.03 ^b,C^	355.87 ± 0.50 ^a,A^	4021.63 ± 0.55 ^a,A^
M_4_72h	6731.87 ± 0.88 ^b,I^	2213.98 ± 0.31 ^b,C^	671.09 ± 0.88 ^b,B^	421.78 ± 0.69 ^b,B^	4128.09 ± 0.62 ^b,B^
M_4_120h	8813.78 ± 0.81 ^c,L^	2119.76 ± 0.59 ^a,B^	653.87 ± 0.49 ^a,A^	474.44 ± 0.74 ^c,C^	4297.07 ± 0.88 ^c,D^
M_5_24h	7701.96 ± 0.33 ^c,K^	2032.41 ± 0.44 ^a,A^	1098.23 ± 0.24 ^a,E^	491.27 ± 0.93 ^a,D^	4461.66 ± 0.67 ^a,E^
M_5_72h	6325.09 ± 0.77 ^b,G^	2540.87 ± 0.26 ^b,G^	1051.88 ± 0.31 ^b,D^	551.09 ± 0.46 ^b,F^	4786.09 ± 0.29 ^b,G^
M_6_120h	5788.88 ± 0.39 ^a,D^	2502.78 ± 0.59 ^b,F^	1130.40 ± 0.82 ^c,G^	694.95 ± 0.88 ^c,J^	4854.43 ± 0.44 ^c,H^
M_6_24h	19812.71 ± 0.50 ^c,Q^	4096.79 ± 0.15 ^c,R^	1543.05 ± 0.30 ^c,O^	708.92 ± 0.99 ^c,K^	6574.61 ± 0.59 ^c,Q^
M_6_72h	16234.67 ± 0.31 ^b,P^	3245.10 ± 0.99 ^b,K^	1342.09 ± 0.22 ^b,J^	623.09 ± 1.50 ^b,H^	5032.90 ± 0.22 ^b,J^
M_6_120h	9734.41 ± 0.91 ^a,M^	2885.98 ± 0.59 ^a,I^	1228.76 ± 0.11 ^a,H^	539.67 ± 1.43 ^a,E^	4874.64 ± 0.49 ^a,I^

Mean values of three different determinations followed by standard deviation; M_1_–sirloin marinated only with salt and pepper; M_2_–sirloin marinated with olive oil, rosemary, salt and pepper; M_3_–sirloin marinated with pumpkin oil, oregano, salt and pepper; M_4_–sirloin marinated with sunflower oil, thyme, salt and pepper; M_5_–sirloin marinated with walnut oil, basil, salt and pepper; M_6_–sirloin marinated with sesame oil, ginger, salt and pepper. Values not sharing the same small letter in a column indicate significant difference between the same sample at different moments: 24 h, 72 h, 120 h. Values not sharing the same capital letters in a row indicate the significant difference between all samples at different moments: 24 h, 72 h, 120 h.

**Table 7:** Texture profile analysis for marinated samples.

Samples	Hardness Cycle 1 [N]	Resilience [mJ]	Hardness Cycle 2 [N]	Cohesiveness [n.a.]	Gumminess [N]	Chewiness [N]
M_1_M24h	1019 ± 0.22 ^c,M^	0.17 ± 0.03 ^a,BC^	883 ± 1.23 ^c,L^	0.36 ± 0.02 ^a,BC^	469 ± 1.34 ^c,K^	66.5 ± 1.34 ^c,GH^
M_1_M72h	997 ± 0.45 ^b,L^	0.16 ± 0.04 ^a,B^	495 ± 0.78 ^b,F^	0.39 ± 0.03 ^ab,BCD^	425 ± 1.56 ^b,I^	63 ± 0.67 ^b,G^
M_1_M120h	985 ± 0.89 ^a,K^	0.16 ± 0.03 ^a,B^	440 ± 1.45 ^a,CE^	0.42 ± 0.11 ^b,BCDE^	399 ± 1.03 ^a,H^	57.0 ± 0.89 ^a,EF^
M_2_M24h	1100 ± 1.24 ^c,N^	0.21 ± 0.02 ^a,BCDE^	612 ± 1.89 ^c,J^	0.41 ± 0.22 ^a,BCDE^	450 ± 0.89 ^c,J^	61.7 ± 1.02 ^c,FG^
M_2_M72h	697 ± 0.76 ^b,F^	0.22 ± 0.02 ^a,CDE^	545 ± 1.03 ^b,G^	0.55 ± 0.02 ^b,EFG^	320 ± 0.90 ^b,F^	53.02 ± 0.76 ^b,E^
M_2_M120h	389 ± 1.67 ^a,A^	0.24 ± 0.12 ^a,EF^	435 ± 0.89 ^a,BCD^	0.69 ± 0.04 ^c,G^	267 ± 1.45 ^a,C^	39.5 ± 0.34 ^a,C^
M_3_M24h	1301 ± 0.88 ^c,P^	0.10 ± 0.03 ^a,A^	1290 ± 0.77 ^c,O^	0.17 ± 0.02 ^a,A^	400 ± 1.52 ^a,H^	46.2 ± 0.59 ^a,D^
M_3_M72h	734 ± 0.45 ^b,G^	0.19 ± 0.02 ^a,BCDE^	765 ± 0.65 ^b,K^	0.20 ± 0.03 ^ab,A^	575 ± 1.09 ^b,L^	73.7 ± 0.52 ^c,I^
M_3_M120h	656 ± 0.27 ^a,E^	0.23 ± 0.02 ^b,DE^	565 ± 0.45 ^a,I^	0.28 ± 0.02 ^b,AB^	580 ± 0.98 ^c,M^	71.10 ± 1.45 ^b,HI^
M_4_M24h	1109 ± 0.39 ^c,O^	0.18 ± 0.04 ^a,BCD^	1023 ± 0.78 ^c,N^	0.28 ± 0.03 ^a,AB^	772 ± 0.45 ^c,O^	124.90 ± 0.89 ^c,J^
M_4_M72h	879 ± 1.04 ^b,I^	0.23 ± 0.04 ^ab,DE^	767 ± 0.44 ^b,K^	0.35 ± 0.04 ^b,BC^	603 ± 0.63 ^b,N^	57.90 ± 0.95 ^b,EF^
M_4_M120h	469 ± 1.87 ^a,B^	0.29 ± 0.02 ^b,F^	340 ± 0.67 ^a,A^	0.47 ± 0.02 ^c,CDEF^	325 ± 1.45 ^a,G^	47.50 ± 0.87 ^a,D^
M_5_M24h	989 ± 1.02 ^c,K^	0.17 ± 0.02 ^a,BC^	435 ± 0.89 ^a,BC^	0.35 ± 0.03 ^a,BC^	253 ± 1.39 ^b,B^	29.70 ± 0.40 ^c,B^
M_5_M72h	514 ± 0.99 ^b,D^	0.18 ± 0.03 ^a,BCD^	421 ± 0.90 ^b,F^	0.45 ± 0.12 ^b,CDEF^	283 ± 1.55 ^c,E^	24.50 ± 1.45 ^b,A^
M_5_M120h	509 ± 0.67 ^a,C^	0.18 ± 0.02 ^a,BCD^	410 ± 0.34 ^c,P^	0.58 ± 0.05 ^c,FG^	183 ± 1.43 ^a,A^	20.50 ± 0.50 ^a,A^
M_6_M24h	1487 ± 0.45 ^c,Q^	0.17 ± 0.04 ^b,BC^	987 ± 0.55 ^c,M^	0.29 ± 0.02 ^a,AB^	429 ± 1.30 ^c,I^	56.90 ± 1.43 ^c,EF^
M_6_M72h	964 ± 0.44 ^b,J^	0.10 ± 0.02 ^a,A^	551 ± 0.90 ^b,H^	0.45 ± 0.03 ^b,CDEF^	273 ± 1.65 ^b,D^	45.91 ± 1.08 ^b,D^
M_6_M120h	792 ± 0.67 ^a,H^	0.11 ± 0.05 ^a,A^	432 ± 0.89 ^a,B^	0.52 ± 0.02 ^c,DEF^	255 ± 1.02 ^a,B^	39.72 ± 1.34 ^a,C^

Mean values of three different determinations followed by standard deviation; M_1_M–sirloin marinated only with salt and pepper; M_2_M–sirloin marinated with olive oil, rosemary, salt and pepper; M_3_M–sirloin marinated with pumpkin oil, oregano, salt and pepper; M_4_M–sirloin marinated with sunflower oil, thyme, salt and pepper; M_5_M-sirloin marinated with walnut oil, basil, salt and pepper; M_6_M-sirloin marinated with sesame oil, ginger, salt and pepper; Values not sharing the same small letter in a column indicate significant difference between the same sample at different moments: 24 h, 72, 120 h. Values not sharing the same capital letters in a row indicate the significant difference between all samples at different moments: 24 h, 72 h, 120 h.

**Table 8:** pH and water loss of the cooked meat samples.

	pH			Water Loss (%)		
Samples	24 h	72 h	120 h	24 h	72 h	120 h
M_1_T (control)	6.03 ± 0.02 ^a,B^	5.94 ± 0.01 ^ab,B^	5.87 ± 0.02 ^aBC^	23.53 ± 0.02 ^abB^	24.32 ± 0.01 ^bD^	23.33 ± 0.04 ^aC^
M_2_T	5.72 ± 0.02 ^a,bA^	5.54 ± 0.01 ^a,A^	5.37 ± 0.02 ^aA^	22.07 ± 0.03 ^cA^	19.02 ± 0.04 ^abA^	18.10 ± 0.05 ^aA^
M_3_T	6.02 ± 0.03 ^a,B^	5.91 ± 0.02 ^ab,B^	6.0 ± 0.03 ^aC^	23.33 ± 0.04 ^cB^	21.59 ± 0.04 ^abBC^	20.29 ± 0.03 ^aB^
M_4_T	6.23 ± 0.02 ^a,CD^	6.03 ± 0.04 ^a,B^	5.90 ± 0.05 ^abBC^	24.12 ± 0.02 ^cB^	20.38 ± 0.03 ^abAB^	19.40 ± 0.04 ^aAB^
M_5_T	6.30 ± 0.02 ^a,bD^	5.89 ± 0.02 ^a,B^	5.72 ± 0.03 ^aB^	23.65 ± 0.05 ^cB^	21.83 ± 0.01 ^abBC^	20.10 ± 0.05 ^aB^
M_6_T	6.11 ± 0.03 ^ab,BC^	6.01 ± 0.05 ^ab,B^	5.42 ± 0.04 ^aA^	26.49 ± 0.03 ^cC^	22.93 ± 0.02 ^abCD^	21.05 ± 0.05 ^aB^

Mean values of three different determinations followed by standard deviation; M_1_T–cooked sirloin marinated only with salt and pepper; M_2_T–cooked sirloin marinated with olive oil, rosemary, salt and pepper; M_3_T–cooked sirloin marinated with pumpkin oil, oregano, salt and pepper; M_4_T–cooked sirloin marinated with sunflower oil, thyme, salt and pepper; M_5_T-cooked sirloin marinated with walnut oil, basil, salt and pepper; M_6_T-cooked sirloin marinated with sesame oil, ginger, salt and pepper. Values not sharing the same small letter in a column indicate significant difference between the same sample at different moments: 24 h, 72 h, 120 h. Values not sharing the same capital letters in a row indicate the significant difference between all samples at different moments: 24 h, 72 h, 120 h.

**Table 9:** Organic acid content of cooked samples.

Samples	Oxalic (μg/g)	Citric (μg/g)	Tartaric (μg/g)	Malic (μg/g)	Lactic (μg/g)
M_1_T24h	10121.68 ± 1.45 ^bN^	668.60 ± 0.55 ^bH^	1329.18 ± 0.77 ^aK^	731.08 ± 0.99 ^bP^	2760.52 ± 1.09 ^cO^
M_1_T72h	11023.89 ± 1.09 ^cR^	643.22 ± 0.98 ^aG^	1345.66 ± 0.89 ^bL^	723.09 ± 0.78 ^aO^	2622.09 ± 0.80 ^bA^
M_1_T120h	5518.32 ± 1.66 ^aH^	690.57 ± 0.88 ^cI^	1676.28 ± 0.61 ^cN^	740.82 ± 0.71 ^cQ^	2255.24 ± 2.11 ^aI^
M_2_T24h	10715.90 ± 0.98 ^cP^	2364.58 ± 0.67 ^cE^	1144.40 ± 0.89 ^bD^	537.46 ± 0.89 ^bH^	5976.65 ± 1.33 ^aQ^
M_2_T72h	7578.92 ± 0.77 ^bL^	2121.33 ± 0.50 ^aB^	1133.22 ± 0.90 ^aC^	520.98 ± 1.06 ^aF^	6052.99 ± 0.89 ^bL^
M_2_T120h	5401.18 ± 0.90 ^aG^	2210.75 ± 0.77 ^bC^	1296.50 ± 0.81 ^cI^	621.34 ± 1.09 ^cJ^	6189.03 ± 0.55 ^cC^
M_3_T24h	6346.07 ± 0.67 ^cK^	2760.58 ± 0.49 ^bK^	1219.00 ± 0.31 ^cH^	726.98 ± 1.89 ^cOP^	4982.52 ± 0.41 ^cG^
M_3_T72h	5341.11 ± 1.45 ^bQ^	2766.22 ± 0.67 ^cL^	1132.44 ± 0.38 ^aC^	702.34 ± 0.90 ^bN^	4789.02 ± 1.09 ^aE^
M_3_T120h	4114.87 ± 1.76 ^aE^	2716.45 ± 0.39 ^aJ^	1152.87 ± 1.09 ^bE^	683.91 ± 0.78 ^aM^	4859.87 ± 0.88 ^bF^
M_4_T24h	10305.11 ± 0.55 ^bO^	3145.78 ± 0.88 ^cQ^	1161.60 ± 0.56 ^aF^	500.49 ± 0.12 ^aE^	5944.52 ± 0.67 ^cP^
M_4_T72h	5231.11 ± 0.45 ^aF^	3012.33 ± 0.56 ^bO^	1322.09 ± 0.88 ^bJ^	577.34 ± 0.89 ^cI^	5633.09 ± 0.45 ^bM^
M_4_T120h	2170.94 ± 0.43 ^cC^	2980.33 ± 1.09 ^aM^	1572.07 ± 0.49 ^cM^	540.51 ± 0.55 ^bK^	5340.01 ± 0.33 ^aK^
M_5_T24h	5802.18 ± 0.33 ^cI^	1966.85 ± 1.98 ^aA^	952.42 ± 0.22 ^aA^	400.96 ± 0.39 ^cC^	4256.16 ± 0.55 ^aB^
M_5_T72h	5550.13 ± 0.85 ^bB^	2544.11 ± 0.99 ^bF^	1123.48 ± 0.23 ^bB^	350.77 ± 0.67 ^bB^	4731.87 ± 0.61 ^bD^
M_5_T120h	3782.82 ± 0.62 ^aD^	3084.21 ± 0.78 ^cP^	1133.10 ± 0.2 1^bcC^	293.11 ± 0.77 ^aA^	5195.30 ± 0.43 ^cH^
M_6_T24h	844.12 ± 1.03 ^cM^	2253.07 ± 0.67 ^aD^	1133.16 ± 0.33 ^bC^	482.04 ± 0.89 ^aD^	5258.39 ± 0.18 ^aI^
M_6_T72h	435.77 ± 1.33 ^aA^	2766.19 ± 0.71 ^bL^	1126.89 ± 0.88 ^aB^	533.22 ± 1.34 ^bG^	5309.27 ± 0.73 ^bJ^
M_6_T120h	5882.30 ± 1.02 ^bJ^	2996.77 ± 0.66 ^cN^	1191.59 ± 0.23 ^cG^	574.36 ± 1.09 ^cL^	5691.07 ± 0.64 ^cN^

Mean values of three different determinations followed by standard deviation; M_1_T–cooked sirloin marinated only with salt and pepper; M_2_T–cooked sirloin marinated with olive oil, rosemary, salt and pepper; M_3_T–cooked sirloin marinated with pumpkin oil, oregano, salt and pepper; M_4_T–cooked sirloin marinated with sunflower oil, thyme, salt and pepper; M_5_T-sirloin marinated with walnut oil, basil, salt and pepper; M6 M_6_T-sirloin marinated with sesame oil, ginger, salt and pepper. Values not sharing the same small letter in a row indicate significant difference between the same sample at different moments: 24 h, 72 h, 120 h. Values not sharing the same capital letters in a row indicate the significant difference between all samples at different moments: 24 h, 72 h, 120 h.

**Table 10:** Texture profile analysis of cooked meat samples.

Samples	Hardness Cycle 1 [N]	Resilience [mJ]	Hardness Cycle 2 [N]	Cohesiveness [n.a.]	Gumminess [N]	Chewiness [N]
M_1_T24h	1588 ± 1.66 ^c,Q^	0.26 ± 0.01 ^a,BCDEF^	1211 ± 1.01 ^c,N^	0.54 ± 0.02 ^ab,ABC^	953 ± 1.09 ^c,P^	125.1 ± 1.04 ^c,J^
M_1_T72h	1201 ± 1.23 ^b,L^	0.22 ± 0.02 ^a,ABC^	1001 ± 0.89 ^b,M^	0.55 ± 0.01 ^b,ABC^	821 ± 0.99 ^b,M^	93.1 ± 0.88 ^b,H^
M_1_T120h	993 ± 0.99 ^a,J^	0.23 ± 0.03 ^a,ABCD^	888 ± 0.77 ^a,K^	0.45 ± 0.12 ^a,AB^	677 ± 0.87 ^a,I^	87.22 ± 0.44 ^a,G^
M_2_T24h	1005 ± 1.45 ^c,K^	0.23 ± 0.02 aABCD	989 ± 0.35 ^c,L^	0.56 ± 0.03 ^a,ABC^	779 ± 0.56 ^c,K^	112.3 ± 0.72 ^c,I^
M_2_T72h	864 ± 0.99 ^b,F^	0.37 ± 0.01 ^ab,GH^	565 ± 0.67 ^b,B^	0.77 ± 0.08 ^ab,EF^	567 ± 1.89 ^b,G^	62.70 ± 1.67b ^d,E^
M_2_T120h	392 ± 0.88 ^a,A^	0.48 ± 0.03 ^b,I^	324 ± 0.42 ^a,A^	0.89 ± 0.02 ^c,F^	257 ± 1.02 ^a,A^	44.02 ± 0.99 ^a,A^
M_3_T24h	1255 ± 1.45 ^c,N^	0.28 ± 0.02 ^a,CDEFG^	1345 ± 0.98 ^c,O^	0.49 ± 0.01 ^a,ABC^	841 ± 1.09 ^c,N^	76.11 ± 1.76 ^c,F^
M_3_T72h	985 ± 1.98 ^b,I^	0.33 ± 0.01 ^ab,DFGH^	799 ± 1.06 ^b,I^	0.55 ± 0.02 ^ab,ABC^	765 ± 0.88 ^b,J^	67.09 ± 0.55 ^b,E^
M_3_T120h	679 ± 0.99 ^a,C^	0.39 ± 0.02 ^b,HI^	670 ± 1.00 ^a,D^	0.59 ± 0.03 ^b,CD^	348 ± 0.76 ^a,B^	50.40 ± 0.99 ^a,B^
M_4_T24h	1455 ± 0.78 ^c,O^	0.17 ± 0.03 ^a,AB^	1400 ± 0.87 ^c,Q^	0.43 ± 0.04 ^ab,A^	822 ± 0.64 ^c,M^	109.32 ± 0.72 ^c,I^
M_4_T72h	1002 ± 0.76 ^b,K^	0.23 ± 0.04 ^ab,ABCDE^	988 ± 0.56 ^b,L^	0.49 ± 0.02 ^a,ABC^	793 ± 0.67 ^b,L^	85.91 ± 1.05 ^b,G^
M_4_T120h	823 ± 0.33 ^a,E^	0.37 ± 0.10 ^b,GH^	753 ± 0.51 ^a,G^	0.58 ± 0.11 ^a,BCD^	621 ± 0.88 ^a,H^	75.40 ± 0.77 ^a,F^
M_5_T24h	1246 ± 0.45 ^c,M^	0.15 ± 0.01 ^a,A^	1358 ± 0.88 ^c,P^	0.58 ± 0.03 ^a,BCD^	1017 ± 0.45 ^c,Q^	109.91 ± 0.53 ^c,I^
M_5_T72h	899 ± 0.89 ^b,G^	0.19 ± 0.02 ^ab,ABC^	686 ± 0.72 ^b,E^	0.70 ± 0.02 ^b,DE^	455 ± 1.35 ^b,F^	61.72 ± 0.33 ^b,D^
M_5_T120h	583 ± 0.91 ^a,B^	0.33 ± 0.03 ^c,FGH^	662 ± 1.02 ^a,C^	0.77 ± 0.12 ^c,EF^	422 ± 1.95 ^a,D^	53.43 ± 0.71 ^a,BC^
M_6_T24h	1489 ± 0.88 ^c,P^	0.21 ± 0.02 ^a,ABC^	765 ± 1.07 ^b,H^	0.50 ± 0.03 ^a,ABC^	899 ± 1.99 ^c,O^	78.11 ± 0.66 ^c,F^
M_6_T72h	951 ± 0.72 ^b,H^	0.25 ± 0.01 ^a,BCDEF^	734 ± 0.88 ^a,F^	0.55 ± 0.02 ^ab,ABC^	414 ± 0.67 ^a,C^	56.80 ± 0.73 ^b,C^
M_6_T120h	687 ± 0.81 ^a,D^	0.34 ± 0.01 ^ab,FGH^	839 ± 0.94 ^c,J^	0.61 ± 0.01 ^b,CD^	432 ± 1.01 ^b,E^	51.50 ± 0.79 ^a,B^

Mean values of three different determinations followed by standard deviation; M_1_T–cooked sirloin marinated only with salt and pepper; M_2_T cooked sirloin marinated with olive oil, rosemary, salt and pepper; M_3_T–cooked sirloin marinated with pumpkin oil, oregano, salt and pepper; M_4_T–cooked sirloin marinated with sunflower oil, thyme, salt and pepper; M_5_T-sirloin marinated with walnut oil, basil, salt and pepper; M_6_T-sirloin marinated with sesame oil, ginger, salt and pepper. Values not sharing the same small letter in a row indicate significant difference between the same sample at different moments: 24 h, 72 h, 120 h. Values not sharing the same capital letters in a row indicate the significant difference between all samples at different moments: 24 h, 72 h, 120 h.

**Table 11:** Hedonic scores for analysed samples.

Sample	Colour	Aroma	Tenderness	Juiciness	Taste and Flavour	Overall Appreciation
M_1_T24h	7.47 ± 1.31 ^c,EFG^	6.93 ± 1.60 ^a,A^	6.27 ± 0.98 ^a,A^	6.27 ± 0.98 ^a,AB^	6.60 ± 1.28 ^a,ABC^	6.57 ± 1.10 ^a,A^
M_1_T72h	6.43 ± 1.38 ^b,BCD^	6.90 ± 1.67 ^a,A^	6.97 ± 1.19 ^ab,AB^	6.93 ± 1.05 ^b,C^	7.00 ± 1.34 ^a,bABCD^	7.10 ± 1.37 ^ab,AB^
M_1_T120h	5.60 ± 1.37 ^a,AB^	7.83 ± 1.23 ^b,BCDEFG^	6.87 ± 1.04 ^ab, AB^	6.87 ± 1.11 ^ab,BC^	7.30 ± 1.39 ^b,ABCD^	7.17 ± 1.42 ^ab,B^
M_2_T24h	7.33 ± 1.21 ^a,DEFG^	7.63 ± 1.40 ^a,BCD^	6.30 ± 0.99 ^a,A^	6.24 ± 0.99 ^a,AB^	6.93 ± 1.28 ^a,ABC^	7.13 ± 1.38 ^a,AB^
M_2_T72h	7.63 ± 1.38 ^ab,FG^	7.93 ± 1.14 ^ab,BCDEFG^	7.10 ± 1.12 ^b, ABC^	7.07 ± 1.23 ^b,C^	7.90 ± 1.01 ^b,D^	7.90 ± 1.03 ^b,C^
M_2_T120h	8.63 ± 1.38 ^c,H^	8.20 ± 0.83 ^b,H^	8.00 ± 0.87 ^c,E^	8.63 ± 0.91 ^c,D^	8.60 ± 0.84 ^c,F^	8.93 ± 0.82 ^c,D^
M_3_T24h	7.17 ± 1.21 ^bc,DEFG^	7.67 ± 1.40 ^a,BCDE^	6.27 ± 0.91 ^a,A^	6.30 ± 0.95 ^a,AB^	7.27 ± 1.26 ^a,ABC^	7.13 ± 1.28 ^a,AB^
M_3_T72h	6.80 ± 1.42 ^b,DEFG^	7.77 ± 1.25 ^a,BCDEFG^	6.83 ± 1.05 ^ab, AB^	6.87 ± 1.04 ^ab,ABC^	7.77 ± 1.01 ^ab,ABCD^	7.87 ± 0.97 ^a,bC^
M_3_T120h	5.80 ± 1.40 ^a,ABC^	8.07 ± 0.78 ^b,GH^	7.83 ± 0.91 ^b,BC^	7.90 ± 0.88 ^c,D^	8.10 ± 0.92 ^b,EF^	8.13 ± 0.90 ^c,C^
M_4_T24h	7.73 ± 1.11 ^a,GH^	7.77 ± 1.30 ^a,BCDEFG^	6.17 ± 0.79 ^a,A^	6.40 ± 0.97 ^a,A^	7.13 ± 1.22 ^a,ABCD^	7.17 ± 1.23 ^a,B^
M_4_T72h	6.57 ± 1.48 ^b,CDE^	7.97 ± 1.13 ^a,EFGH^	6.97 ± 1.13 ^ab, AB^	7.00 ± 1.17 ^b,C^	7.87 ± 1.01 ^ab,CD^	7.77 ± 1.04 ^ab,C^
M_4_T120h	5.40 ± 1.45 ^c,A^	8.07 ± 0.81 ^ab,GH^	7.87 ± 1.01 ^c,BC^	7.87 ± 0.94 ^b,cD^	8.00 ± 0.91 ^b,EF^	8.03 ± 0.89 ^c,C^
M_5_T24h	7.60 ± 1.25 ^c,FG^	7.70 ± 1.34 ^a,BCDEF^	6.24 ± 0.99 ^a,A^	6.20 ± 1.00 ^a,AB^	7.23 ± 1.28 ^a,A^	7.13 ± 1.25 ^a,AB^
M_5_T72h	6.73 ± 1.41 ^b,DEF^	7.73 ± 1.34 ^a,BCDEFG^	7.17 ± 1.18 ^ab,BC^	7.07 ± 1.01 ^ab,C^	7.83 ± 0.95 ^ab,D^	7.80 ± 1.00 ^ab,C^
M_5_T120h	5.57 ± 1.28 ^a,AB^	8.03 ± 0.89 ^ab,FGH^	7.90 ± 0.99 ^b,CD^	7.87 ± 1.04 ^bc,D^	8.13 ± 0.78 ^c,EF^	8.13 ± 0.78 ^b,C^
M_6_T24h	7.20 ± 1.42 ^c,DEFG^	7.63 ± 1.52 ^a,BC^	6.23 ± 0.94 ^a,A^	6.20 ± 0.92 ^a,AB^	7.07 ± 1.01 ^a,AB^	6.90 ± 1.24 ^a,AB^
M_6_T72h	6.73 ± 1.34 ^b,DEF^	7.60 ± 1.35 ^a,B^	6.97 ± 1.19 ^ab,AB^	6.97 ± 1.19 ^ab,C^	7.80 ± 0.96 ^ab,ACD^	7.83 ± 1.02 ^b,C^
M_6_T120h	5.57 ± 1.48 ^a,AB^	8.00 ± 0.83 ^ab,EFGH^	7.80 ± 1.00 ^c,CD^	7.80 ± 1.06 ^c,D^	8.10 ± 0.88 ^b,E^	8.07 ± 0.91 ^c,C^

Values represent hedonic scores calculated as mean ± SD (*n* = 30); M_1_T cooked sirloin marinated only with salt and pepper; M_2_T–cooked sirloin marinated with olive oil, rosemary, salt and pepper; M_3_T–cooked sirloin marinated with pumpkin oil, oregano, salt and pepper; M_4_T–cooked sirloin marinated with sunflower oil, thyme, salt and pepper; M_5_T-sirloin marinated with walnut oil, basil, salt and pepper; M_6_T-sirloin marinated with sesame oil, ginger, salt and pepper; Values not sharing the same small letter in a row indicate significant difference between the same sample at different moments: 24 h, 72 h, 120 h. Values not sharing the same capital letters in a row indicate the significant difference between all samples at different moments: 24 h, 72 h, 120 h.

## Supplementary Materials

The following are available online at https://www.mdpi.com/article/10.3390/foods10092012/s1, Table S1: Marinated sirloin recipes, Table S2: Phenolics profile of marinated samples, Table S3: Phenolics profile of cooked samples.
