# Supplementary material for: Influence of Marination with Aromatic Herbs and Cold Pressed Oils on Black Angus Beef Meat
Source: Foods. 2021 Aug 27;10(9):2012. doi: 10.3390/foods10092012 (PMC8468897; doi:10.3390/foods10092012)
Supplement: Supplementary file 1 [file foods-10-02012-s001.zip › foods-1356928-supplementary.pdf]

Table S1:Marinated sirloin recipes

| **Marinade ingredients** | **UM** | **Marinade 1 ($M_1$) control** | **Marinade 2 ($M_2$)** | **Marinade 3 ($M_3$)** | **Marinade 4 ($M_4$)** | **Marinade 5 ($M_5$)** | **Marinade 6 ($M_6$)** |
|---|---|---|---|---|---|---|---|
| Rosemary | kg/kg | | 0.014 | | | | |
| Olive oil | L/kg | | 0.048 | | | | |
| Oregano | kg/kg | | | 0.014 | | | |
| Pumpkin oil | L/kg | | | 0.048 | | | |
| Thyme | kg/kg | | | | 0.014 | | |
| Sunflower oil | L/kg | | | | 0.048 | | |
| Basil | kg/kg | | | | | 0.014 | |
| Walnut oil | L/kg | | | | | 0.048 | |
| Ginger | kg/kg | | | | | | 0.014 |
| Sesame oil | L/kg | | | | | | 0.048 |
| Salt | % | 2 | 2 | 2 | 2 | 2 | 2 |
| Black ground pepper | % | 0.50 | 0.50 | 0.50 | 0.50 | 0.50 | 0.50 |

Table S2: Phenolics profile of marinated samples

| Compound | $M_1$ 24h | $M_1$ 72h | $M_1$ 120h | $M_2$ 24h | $M_2$ 72h | $M_2$ 120h | $M_3$ 24h | $M_3$ 72h | $M_3$ 120h | $M_4$ 24h | $M_4$ 72h | $M_4$ 120h | $M_5$ 24h | $M_5$ 72h | $M_6$ 120h | $M_6$ 24h | $M_6$ 72h | $M_6$ 120h |
|---|---|---|---|---|---|---|---|---|---|---|---|---|---|---|---|---|---|---|
| Flavones | | | | | | | | | | | | | | | | | | |
| HLG | n.d | n.d | n.d | 21.20±023$^{a}$ | 21.33±0.45$^{a}$ | 21.45±0.78$^{a}$ | n.d | n.d | n.d | n.d | n.d | n.d | n.d | n.d | n.d | n.d | n.d | n.d |
| NP | n.d | n.d | n.d | 48.18±0.88$^{c}$ | 35.23±0.33$^{b}$ | 20.47±0.67$^{a}$ | n.d | n.d | n.d | n.d | n.d | n.d | n.d | n.d | n.d | n.d | n.d | n.d |
| LG | n.d | n.d | n.d | n.d | n.d | n.d | 30.93±0.4$^{ab}$ | 29.03±0.33$^{a}$ | 29.01±0.7$^{a}$ | 30.05±0.8$^{ab}$ | 29.09±0.77$^{a}$ | 29.04±0.37$^{a}$ | n.d | n.d | n.d | n.d | n.d | n.d |
| PL | n.d | n.d | n.d | 30.64±0.78$^{b}$ | 29.32±0.44$^{ab}$ | 28.46±0.88$^{a}$ | n.d | n.d | n.d | n.d | n.d | n.d | n.d | n.d | n.d | n.d | n.d | n.d |
| LAG | n.d | n.d | n.d | 23.26±0.99$^{c}$ | 22.27±0.77$^{ab}$ | 21.99±0.85$^{a}$ | n.d | n.d | n.d | n.d | n.d | n.d | n.d | n.d | n.d | n.d | n.d | n.d |
| LA | n.d | n.d | n.d | n.d | n.d | n.d | 4.99±0.77$^{a}$ | 4.56±0.99$^{a}$ | 5.10±0.89$^{a}$ | 15.73±0.88$^{b}$ | 14.22±0.76$^{a}$ | 15.05 ±0.98$^{ab}$ | n.d | n.d | n.d | n.d | n.d | n.d |
| CRM | n.d | n.d | n.d | 59.61±0.89$^{c}$ | 53.76±1.23$^{b}$ | 51.82±1.67$^{a}$ | n.d | n.d | n.d | n.d | n.d | n.d | n.d | n.d | n.d | n.d | n.d | n.d |
| HP | n.d | n.d | n.d | n.d | n.d | n.d | 21.83±0.89$^{c}$ | 19.23±0.77$^{ab}$ | 18.76±0.8$^{a}$ | n.d | n.d | n.d | n.d | n.d | n.d | n.d | n.d | n.d |
| AP | n.d | n.d | n.d | n.d | n.d | n.d | 16.87±0.89$^{c}$ | 11.77±0.85$^{ab}$ | 10.13±1.1$^{a}$ | n.d | n.d | n.d | n.d | n.d | n.d | n.d | n.d | n.d |
| LG2 | n.d | n.d | n.d | n.d | n.d | n.d | 9.40±0.56$^{ab}$ | 8.81±0.45$^{a}$ | 8.98±0.23$^{a}$ | n.d | n.d | n.d | n.d | n.d | n.d | n.d | n.d | n.d |
| LGRS | n.d | n.d | n.d | 62.04±1.23$^{c}$ | 55.33±0.99$^{b}$ | 53.02±1.35$^{a}$ | n.d | n.d | n.d | n.d | n.d | n.d | n.d | n.d | n.d | | | |
| Hydroxycinnamic acids | | | | | | | | | | | | | | | | | | |
| DIFQ | 22.44±1.09$^{c}$ | 20.74±0.78$^{ab}$ | 19.17±0.56$^{a}$ | n.d | n.d | n.d | 12.90±0.23$^{b}$ | 11.99±0.56$^{a}$ | 11.22±0.8$^{a}$ | 32.13±0.78$^{b}$ | 31.09±0.59$^{a}$ | 30.70±1.01$^{a}$ | n.d | n.d | n.d | n.d | n.d | n.d |
| CHG | n.d | n.d | n.d | n.d | n.d | n.d | 17.22±0.49$^{ab}$ | 16.99±0.44$^{a}$ | 16.27±0.7$^{a}$ | n.d | n.d | n.d | n.d | n.d | n.d | n.d | n.d | n.d |
| RSM | n.d | n.d | n.d | n.d | n.d | n.d | 4.73±0.11$^{ab}$ | 4.21±0.67$^{a}$ | 3.91±0.55$^{a}$ | 9.32±0.34$^{b}$ | 7.01±0.11$^{a}$ | 7.98±0.22$^{a}$ | 27.73±1.23$^{c}$ | 25.77±0.67$^{ab}$ | 23.95±0.88$^{a}$ | n.d | n.d | n.d |
| CHA | n.d | n.d | n.d | n.d | n.d | n.d | n.d | n.d | n.d | n.d | n.d | n.d | 17.42±0.55$^{ab}$ | 16.99±0.78$^{a}$ | 16.53±0.88$^{a}$ | n.d | n.d | n.d |
| Phenolic terpene | | | | | | | | | | | | | | | | | | |
| CRN | n.d | n.d | n.d | 52.30±1.56$^{b}$ | 49.52±1.09$^{a}$ | 50.38±0.99$^{a}$ | 39.99±0.56$^{c}$ | 36.77±0.66$^{b}$ | 33.86±0.2$^{a}$ | n.d | n.d | n.d | 30.89±0.33$^{b}$ | 31.53±0.55$^{bc}$ | 27.91±0.59$^{a}$ | n.d | n.d | n.d |
| CAR | n.d | n.d | n.d | 67.59±0.64$^{a}$ | 77.11±0.84$^{b}$ | 88.82±0.87$^{c}$ | n.d | n.d | n.d | n.d | n.d | n.d | n.d | n.d | n.d | n.d | n.d | n.d |
| phenolic acids | | | | | | | | | | | | | | | | | | |
| DHB | 37.84±1.23$^{c}$ | 32.84±0.98$^{b}$ | 2946±0.88$^{a}$ | 9917±1.78$^{c}$ | 6703±1.23$^{b}$ | 54.08±0.99$^{a}$ | 49.27±0.34$^{c}$ | 35.09±1.65$^{b}$ | 19.35±1.9$^{a}$ | 41.18±1.02$^{c}$ | 37.09±1.86$^{b}$ | 24.04±0.77$^{a}$ | 71.337±0.6$^{c}$ | 55.73±0.62$^{a}$ | 58.25±0.27$^{b}$ | 18.93±0.29$^{a}$ | 37.67±1.77$^{b}$ | 42.37±0.89$^{c}$ |
| HB | 184.94±1.0$^{c}$ | 181.08±1.0$^{b}$ | 174.83±1.0$^{c}$ | 205.82±0.98$^{a}$ | 241.09±0.65$^{b}$ | 248.17±0.88$^{c}$ | 201.06±0.67$^{a}$ | 237.99±0.62$^{b}$ | 261.2±0.85$^{c}$ | 248.59±0.63$^{a}$ | 255.94±0.38$^{b}$ | 257.276±0.39b$^{c}$ | 237.88±1.21$^{b}$ | 231.88±0.99$^{a}$ | 242.643±0.23$^{c}$ | 184.47±0.78$^{a}$ | 201.89±0.53$^{b}$ | 223.52±0.49$^{c}$ |
| Tyrosol | | | | | | | | | | | | | | | | | | |

| | | | | | | | | | | | | | | | | | | |
|---|---|---|---|---|---|---|---|---|---|---|---|---|---|---|---|---|---|---|
| HDT | n.d | n.d | n.d | 9.71±0.59[ab] | 9.01±0.89[a] | 9.40±0.25[a] | n.d | n.d | n.d | n.d | n.d | n.d | n.d | n.d | n.d | n.d | n.d | n.d |
| TYR | n.d | n.d | n.d | 13.52±0.52[a] | 14.82±0.77[ab] | 15.85±0.59[b] | n.d | n.d | n.d | n.d | n.d | n.d | n.d | n.d | n.d | n.d | n.d | n.d |
| OLEP | n.d | n.d | n.d | 43.79±0.69[c] | 39.28±0.78[ab] | 38.83±0.31[a] | n.d | n.d | n.d | n.d | n.d | n.d | n.d | n.d | n.d | n.d | n.d | n.d |
| Lignans | | | | | | | | | | | | | | | | | | |
| ISR | n.d | n.d | n.d | n.d | n.d | n.d | n.d | n.d | n.d | 8.58±0.92[ab] | 7.54±0.83[a] | 7.09±0.33[a] | n.d | n.d | n.d | n.d | n.d | n.d |
| SES | n.d | n.d | n.d | n.d | n.d | n.d | n.d | n.d | n.d | n.d | n.d | n.d | n.d | n.d | n.d | 27.91±0.63[a] | 27.32±0.72[a] | 27.44±0.27[a] |
| Hydroxyphenylpropene | | | | | | | | | | | | | | | | | | |
| PRD | | n.d | n.d | n.d | n.d | n.d | n.d | n.d | n.d | n.d | n.d | n.d | n.d | n.d | n.d | 36.83±1.23[ab] | 35.03±0.51[a] | 34.22±1.04[a] |
| GNG | | n.d | n.d | n.d | n.d | n.d | n.d | n.d | n.d | n.d | n.d | n.d | n.d | n.d | n.d | 23.15±0.79[ab] | 22.00±0.99[a] | 22.83±0.76[a] |
| Hydroxybenzaldehide | | | | | | | | | | | | | | | | | | |
| VAN | | n.d | n.d | n.d | n.d | n.d | 4.77±0.39[a] | 4.56±0.88[a] | 4.33±0.91[a] | n.d | n.d | n.d | n.d | n.d | n.d | n.d | n.d | n.d |

Hydroxy-Luteolin-glucoside – HLG; Nepetrin: NP; Luteolin-glucuronide -LG; Plantaginin: PL; Luteolin-acetyl-glucuronide: LAG; Luteolin-glucoside: LA; Cirsimaritin: CRM; Homoplantaginin (Hispidulin-glucoside): HP; Apigenin-glucoside: AP; Luteolin-glucoside2:LG2; Luteolin-glucuronide (Rosemaryic acid): LGRS; Dicaffeoylquinic acid: DIFQ; Chlorogenic acid: CHG; Rosemaryic acid-RSM; Chicoric acid: CHA; Carnosol: CRN; Carnosic acid: CAR; Dihydroxybenzoic acid: DHB; Hydroxybenzoic acid: HB; Hydroxytyrosol: HDT; Tyrosol: TYR; Oleoropein derivative: OLEP; Isolariciresinol: ISR; Sesamin: SES; Paradol: PRD; Gingerol: GNG; Vanilin: VAN

Mean values of three different determinations followed by standard deviation; $M_1$ – sirloin marinated only with salt and pepper; $M_2$ – sirloin marinated with olive oil, rosemary, salt and pepper; $M_3$ – sirloin marinated with pumpkin oil, oregano, salt and pepper; $M_4$ – sirloin marinated with sunflower oil, thyme, salt and pepper; $M_5$-sirloin marinated with walnut oil, basil, salt and pepper; $M_6$-sirloin marinated with sesame oil, ginger, salt and pepper; Values not sharing the same small letter in a row indicate significant difference between the same sample at different moments: 24h, 72, 120h; n.d.–not detected

Table S3:Phenolics profile of cooked samples

| Compounds μg/g | M$_1$T 24h | M$_1$T 72h | M$_1$T 120h | M$_2$T 24h | M$_2$T 72h | M$_2$T 120h | M$_3$T 24h | M$_3$T 72h | M$_3$T 120h | M$_4$T 24h | M$_4$T 72h | M$_4$T 120h | M$_5$T 24h | M$_5$T 72h | M$_6$T 120h | M$_6$T 24h | M$_6$T 72h | M$_6$T 120h |
|---|---|---|---|---|---|---|---|---|---|---|---|---|---|---|---|---|---|---|
| *Flavones* | | | | | | | | | | | | | | | | | | |
| HLG | n.d. | n.d. | n.d. | 3.48±0.23$^{a}$ | 4.03±0.38$^{ab}$ | 5.08±0.43$^{c}$ | n.d. | n.d. | n.d. | n.d. | n.d. | n.d. | n.d. | n.d. | n.d. | n.d. | n.d. | n.d. |
| NP | n.d. | n.d. | n.d. | 14.25±0.47$^{a}$ | 15.78±0.76$^{b}$ | 17.88±0.88$^{c}$ | n.d. | n.d. | n.d. | n.d. | n.d. | n.d. | n.d. | n.d. | n.d. | n.d. | n.d. | n.d. |
| LG | n.d. | n.d. | n.d. | n.d. | n.d. | n.d. | 20.72±0.22$^{a}$ | 24.43±0.67$^{b}$ | 27.99±0.33$^{c}$ | 19.02±0.87$^{a}$ | 19.76±0.56$^{a}$ | 19.6±0.45$^{a}$ | | | | n.d. | n.d. | n.d. |
| PL | n.d. | n.d. | n.d. | 11.93±0.77$^{a}$ | 12.1±0.36$^{ab}$ | 13.21±0.65$^{b}$ | n.d. | n.d. | n.d. | n.d. | n.d. | n.d. | n.d. | n.d. | n.d. | n.d. | n.d. | n.d. |
| LAG | n.d. | n.d. | n.d. | 7.75±0.12a | 7.02±0.34$^{ab}$ | 6.61±0.54$^{a}$ | n.d. | n.d. | n.d. | n.d. | n.d. | n.d. | n.d. | n.d. | n.d. | n.d. | n.d. | n.d. |
| LA | n.d. | n.d. | n.d. | n.d. | n.d. | n.d. | 5.54±0.31$^{a}$ | 5.04±0.55$^{a}$ | 5.93±0.03$^{a}$ | 10.34±0.49$^{a}$ | 10.89±0.67$^{a}$ | 11.02±0.88$^{a}$ | n.d. | n.d. | n.d. | n.d. | n.d. | n.d. |
| CRM | n.d. | n.d. | n.d. | 27.38±0.7$^{a}$ | 34.23±0.67$^{b}$ | 39.872±0.77$^{c}$ | n.d. | n.d. | n.d. | n.d. | n.d. | n.d. | n.d. | n.d. | n.d. | n.d. | n.d. | n.d. |
| HP | n.d. | n.d. | n.d. | n.d. | n.d. | n.d. | 17.94±1.0$^{c}$ | 12.5±0.2$^{ab}$ | 11.56±0.05$^{a}$ | n.d. | n.d. | n.d. | n.d. | n.d. | n.d. | n.d. | n.d. | n.d. |
| AP | n.d. | n.d. | n.d. | n.d. | n.d. | n.d. | 13.69±0.8$^{a}$ | 13.99±0.2$^{a}$ | 14.5±0.55$^{ab}$ | n.d. | n.d. | n.d. | n.d. | n.d. | n.d. | n.d. | n.d. | n.d. |
| LG2 | n.d. | n.d. | n.d. | n.d. | n.d. | n.d. | 8.56±0.03$^{a}$ | 7.55±0.46$^{a}$ | 8.66±0.55$^{a}$ | n.d. | n.d. | n.d. | n.d. | n.d. | n.d. | n.d. | n.d. | n.d. |
| *Hydroxycinnamic acids* | | | | | | | | | | | | | | | | | | |
| DIFQ | 18.5±0.2$^{a}$ | 18.02±0.4$^{a}$ | 17.92±0.5$^{a}$ | n.d. | n.d. | n.d. | 11.22±0.3$^{a}$ | 10.22±0.4$^{a}$ | 10.33±0.1$^{a}$ | 20.01±0.89$^{a}$ | 19.88±0.44$^{a}$ | 20.07±0.31$^{a}$ | n.d. | n.d. | n.d. | n.d. | n.d. | n.d. |
| CHG | n.d. | n.d. | n.d. | n.d. | n.d. | n.d. | 15.00±0.3$^{a}$ | 15.01±0.2$^{a}$ | 14.49±0.56$^{a}$ | n.d. | n.d. | n.d. | n.d. | n.d. | n.d. | n.d. | n.d. | n.d. |
| RSM | n.d. | n.d. | n.d. | 24.45±0.7$^{a}$ | 27.09±0.89$^{b}$ | 29.62±0.33$^{c}$ | 4.26±0.12$^{c}$ | 3.99±0.2$^{ab}$ | 3.04±0.10$^{a}$ | 4.34±0.23$^{a}$ | 4.09±0.12$^{a}$ | 5.08±0.23$^{ab}$ | 23.95±0.67$^{a}$ | 23.00±0.78$^{a}$ | 24.16±0.6$^{ab}$ | n.d. | n.d. | n.d. |
| CHA | n.d. | n.d. | n.d. | n.d. | n.d. | n.d. | n.d. | n.d. | n.d. | n.d. | n.d. | n.d. | 16.44±0.88$^{b}$ | 15.23±0.2$^{ab}$ | 14.76±0.55$^{a}$ | n.d. | n.d. | n.d. |
| *Phenolic terpene* | | | | | | | | | | | | | | | | | | |
| CRN | n.d. | n.d. | n.d. | 35.9±0.88$^{ab}$ | 34.99±0.34$^{a}$ | 35.56±0.65$^{a}$ | 21.61±0.33$^{a}$ | 21.06±0.56$^{a}$ | 21.09±0.33$^{a}$ | 26.13±0.55 | n.d. | n.d. | 26.13±0.35$^{a}$ | 25.21±0.83$^{a}$ | 25.52±0.67$^{a}$ | n.d. | n.d. | n.d. |
| CAR | n.d. | n.d. | n.d. | 67.05±0.89$^{a}$ | 67.33±0.56$^{a}$ | 66.92±0.44$^{a}$ | n.d. | n.d. | n.d. | n.d. | n.d. | n.d. | n.d. | n.d. | n.d. | n.d. | n.d. | n.d. |
| *phenolic acids* | | | | | | | | | | | | | | | | | | |
| DHB | 66.1 ±1.12$^{c}$ | 48.23±0.87$^{b}$ | 28.15±1.34$^{a}$ | 132.7±0.19$^{c}$ | 89.45±0.88$^{b}$ | 75.08±0.67$^{a}$ | 15.29±0.76$^{a}$ | 17.92±1.56$^{b}$ | 19.11±0.45$^{c}$ | 32.92±1.2$^{ab}$ | 27.09±0.89$^{a}$ | 27.91±0.22$^{a}$ | 49.92±0.34$^{c}$ | 43.09±0.11$^{b}$ | 40.70±0.31$^{a}$ | 38.62±0.77$^{a}$ | 45.98±0.81$^{b}$ | 59.96±0.47$^{c}$ |
| HB | 196.1±0.7$^{a}$ | 199.53±0.2$^{b}$ | 210.97±0.4$^{c}$ | 252.99±0.5$^{a}$ | 255.09±0.89 a$^{b}$ | 262.57±0.7±2$^{c}$ | 255.68±0.6$^{a}$ | 267.09±0.3$^{b}$ | 285.47±0.56$^{c}$ | 293.43±0.75$^{c}$ | 290.99±1.22$^{b}$ | 272.38±1.09$^{a}$ | 266.43±1.43$^{b}$ | 260.00±0.56$^{a}$ | 261.91±0.89$^{a}$ | 250.79±0.78$^{a}$ | 273.21±0.54$^{b}$ | 292.54±0.92$^{c}$ |
| *Tyrosol* | | | | | | | | | | | | | | | | | | |
| HDT | n.d. | n.d. | n.d. | 13.34±0.2c | 10.22±0.45$^{b}$ | 7.87±0.33$^{a}$ | n.d. | n.d. | n.d. | n.d. | n.d. | n.d. | n.d. | n.d. | n.d. | n.d. | n.d. | n.d. |
| TYR | n.d. | n.d. | n.d. | 5.67±0.23$^{a}$ | 6.66±0.76$^{ab}$ | 7.69±0.39$^{c}$ | n.d. | n.d. | n.d. | n.d. | n.d. | n.d. | n.d. | n.d. | n.d. | n.d. | n.d. | n.d. |
| OLEP | n.d. | n.d. | n.d. | 28.63±0.3$^{c}$ | 25.09±0.78$^{b}$ | 17.38±0.34$^{a}$ | n.d. | n.d. | n.d. | n.d. | n.d. | n.d. | n.d. | n.d. | n.d. | n.d. | n.d. | n.d. |
| *Lignans* | | | | | | | | | | | | | | | | | | |
| ISR | n.d. | n.d. | n.d. | n.d. | n.d. | n.d. | n.d. | n.d. | n.d. | 3.01±0.45$^{b}$ | 3.09±0.22$^{ab}$ | 2.93±0.31$^{a}$ | n.d. | n.d. | n.d. | n.d. | n.d. | n.d. |
| SES | n.d. | n.d. | n.d. | n.d. | n.d. | n.d. | n.d. | n.d. | n.d. | n.d. | n.d. | n.d. | n.d. | n.d. | n.d. | 19.15±0.23$^{a}$ | 19.05±0.11$^{a}$ | 19.31±0.21$^{a}$ |
| *Hydroxyphenylpropene* | | | | | | | | | | | | | | | | | | |
| PRD | n.d. | n.d. | n.d. | n.d. | n.d. | n.d. | n.d. | n.d. | n.d. | n.d. | n.d. | n.d. | n.d. | n.d. | n.d. | 20.71±0.34$^{a}$ | 19.09±0.21$^{a}$ | 19.99±0.11$^{a}$ |
| *GNG* | n.d. | n.d. | n.d. | n.d. | n.d. | n.d. | n.d. | n.d. | n.d. | n.d. | n.d. | n.d. | n.d. | n.d. | n.d. | 13.521±0.3$^{a}$ | 12.04±0.45$^{a}$ | 13.261±0.7$^{a}$ |
| *Hydroxybenzaldehide* | | | | | | | | | | | | | | | | | | |
| VAN | n.d. | n.d. | n.d. | n.d. | n.d. | n.d. | n.d. | n.d. | n.d. | 2.45±0.56$^{a}$ | 2.33±0.33$^{a}$ | 2.66±0.41$^{a}$ | n.d. | n.d. | n.d. | n.d. | n.d. | n.d. |

Hydroxy-Luteolin-glucoside – HLG; Nepetrin: NP; Luteolin-glucuronide -LG; Plantaginin: PL; Luteolin-acetyl-glucuronide: LAG; Luteolin-glucoside: LA; Cirsimaritin: CRM; Homoplantaginin (Hispidulin-glucoside): HP; Apigenin-glucoside: AP; Luteolin-glucoside2:LG2; Luteolin-glucuronide (Rosemaryic acid): LGRS; Dicaffeoylquinic acid: DIFQ; Chlorogenic acid: CHG; Rosemaryic acid-RSM; Chicoric acid: CHA; Carnosol: CRN; Carnosic acid: CAR; Dihydroxybenzoic acid: DHB; Hydroxybenzoic acid: HB; Hydroxytyrosol: HDT; Tyrosol: TYR; Oleoropein derivative: OLEP; Isolariciresinol: ISR; Sesamin: SES; Paradol: PRD; Gingerol: GNG; Vanilin: VAN; Mean values of three different determinations followed by standard deviation; M$_1$T – cooked sirloin marinated only with salt and pepper; M$_2$T – cooked sirloin marinated with olive oil, rosemary, salt and pepper; M$_3$T – cooked sirloin marinated with pumpkin oil, oregano, salt and pepper; M$_4$T – cooked sirloin marinated with sunflower oil, thyme, salt and pepper; M5-sirloin marinated with walnut oil, basil, salt and pepper; M6-sirloin marinated with sesame oil, ginger, salt and pepper; Values not sharing the same small letter in a row indicate significant difference between the same sample at different moments: 24h, 72h, 120h; n.d.–not detected.
